# Discovery of Unusual Biaryl Polyketides by Activation of a Silent *Streptomyces venezuelae* Biosynthetic Gene Cluster

**DOI:** 10.1002/cbic.201600396

**Published:** 2016-10-13

**Authors:** Anyarat Thanapipatsiri, Juan Pablo Gomez‐Escribano, Lijiang Song, Maureen J. Bibb, Mahmoud Al‐Bassam, Govind Chandra, Arinthip Thamchaipenet, Gregory L. Challis, Mervyn J. Bibb

**Affiliations:** ^1^ Department of GeneticsFaculty of ScienceKasetsart University, 50 Ngamwongwan Road, Ladyao, ChatuchakBangkok10900Thailand; ^2^ Department of Molecular MicrobiologyJohn Innes CentreNorwich Research ParkNorwichNR4 7UHUK; ^3^ Department of ChemistryUniversity of WarwickGibbet Hill RoadCoventryCV4 7ALUK; ^4^ Center for Advanced Studies in Tropical Natural ResourcesNRU–KUKasetsart University50 Ngamwongwan Road, Ladyao, ChatuchakBangkok10900Thailand; ^5^ Department of BioengineeringUniversity of California San Diego9500 Gilman Drive, MC 0412La JollaCA92093-0412USA

**Keywords:** *bldM*, halogenases, large ATP-binding LuxR-like regulator, oxygen heterocycles, polyketides

## Abstract

Comparative transcriptional profiling of a Δ*bldM* mutant of *Streptomyces venezuelae* with its unmodified progenitor revealed that the expression of a cryptic biosynthetic gene cluster containing both type I and type III polyketide synthase genes is activated in the mutant. The 29.5 kb gene cluster, which was predicted to encode an unusual biaryl metabolite, which we named venemycin, and potentially halogenated derivatives, contains 16 genes including one—*vemR*—that encodes a transcriptional activator of the large ATP‐binding LuxR‐like (LAL) family. Constitutive expression of *vemR* in the Δ*bldM* mutant led to the production of sufficient venemycin for structural characterisation, confirming its unusual biaryl structure. Co‐expression of the venemycin biosynthetic gene cluster and *vemR* in the heterologous host *Streptomyces coelicolor* also resulted in venemycin production. Although the gene cluster encodes two halogenases and a flavin reductase, constitutive expression of all three genes led to the accumulation only of a monohalogenated venemycin derivative, both in the native producer and the heterologous host. A competition experiment in which equimolar quantities of sodium chloride and sodium bromide were fed to the venemycin‐producing strains resulted in the preferential incorporation of bromine, thus suggesting that bromide is the preferred substrate for one or both halogenases.

## Introduction

Polyketides are structurally diverse natural products from plants and microorganisms that exhibit a broad range of biological activities.^1^, ^2^, ^3^ They are assembled through a process similar to fatty acid biosynthesis, involving one or more rounds of decarboxylative condensation of an (alkyl) malonyl thioester extender unit with an acyl thioester starter unit. Several distinct classes of polyketide synthases exist; they are differentiated by their architectures and catalytic mechanisms.^4^

The type I PKSs each consist of one or more multifunctional proteins containing various catalytic domains that are used to assemble their metabolic products. With the exception of PKSs that utilise *trans*‐acting acyl transferases,^5^ a type I PKS generally contains a minimum of three domains: a ketosynthase (KS), an acyl transferase (AT) and an acyl carrier protein (ACP). Optional ketoreductase (KR), dehydratase (DH) and enoyl reductase (ER) domains define the degree of β‐carbon processing after each round of chain elongation.^6^ Type I PKSs can be divided into two subgroups.

The first subgroup is termed “modular”; a typical representative consists of a series of modules, each of which contains a set of distinct catalytic domains that are responsible for one round of chain elongation and subsequent β‐carbon modification in the overall chain assembly process. The archetypal example of such PKSs is 6‐deoxyerythronolide B synthase (DEBS), which is involved in the biosynthesis of the antibiotic erythromycin in the actinobacterium *Saccharopolyspora erythraea*.^7^ The second subgroup is termed “iterative” and is exemplified by 6‐methylsalicylic acid synthase (MSAS), found in many fungi.

Type II PKSs each consist of a series of monofunctional enzymes that are used iteratively in the assembly of polycyclic aromatic metabolites (e.g., actinorhodin and tetracycline) and are mainly found in Actinobacteria.^8^, ^9^

Type III PKSs (e.g., chalcone and stilbene synthases) were first identified in plants, but have since been found in many eubacteria.^10^ They are multifunctional enzymes that catalyse elongation of diverse acyl‐CoA starter units with one or more malonyl‐CoA extender units to form poly‐β‐ketoacyl‐CoA intermediates that undergo a range of cyclisation reactions to form diverse aromatic products.^4^, ^7^, ^11^ This type of PKS is exemplified by RppA, which assembles 1,3,6,8‐tetrahydroxynaphthalene (THN) in several *Streptomyces* species.^12^, ^13^, ^14^, ^15^

S*treptomyces* is the largest genus within the Actinobacteria phylum. *Streptomyces* species are a prolific source of antibiotics and other bioactive natural products with a broad range of applications in both medicine and agriculture.^16^ The recent sequencing of a large number of actinobacterial genomes has revealed the presence of numerous gene clusters with the potential to direct the biosynthesis of new specialised metabolites. However, in most cases the metabolic products of such cryptic gene clusters remain to be identified, presumably because they are not expressed under laboratory growth conditions.^17^ Although well known as a producer of chloramphenicol, *Streptomyces venezuelae* ATCC 10712 failed to produce this antibiotic in our laboratory under a range of culture conditions. However, for reasons that we do not yet understand, deletion of *bldM*, which encodes an atypical response regulator required for morphological development,^18^ activated transcription of the chloramphenicol biosynthetic gene cluster.^19^ Here we report the discovery of an unusual biaryl polyketide as the metabolic product of a cryptic polyketide biosynthetic gene cluster that was identified by comparative transcriptional analyses of the wild‐type and Δ*bldM* strains of *S. venezuelae*.

## Results and Discussion

### Identification of a cryptic polyketide biosynthetic gene cluster in *S. venezuelae* and prediction of its biosynthetic product

**Figure 1:**
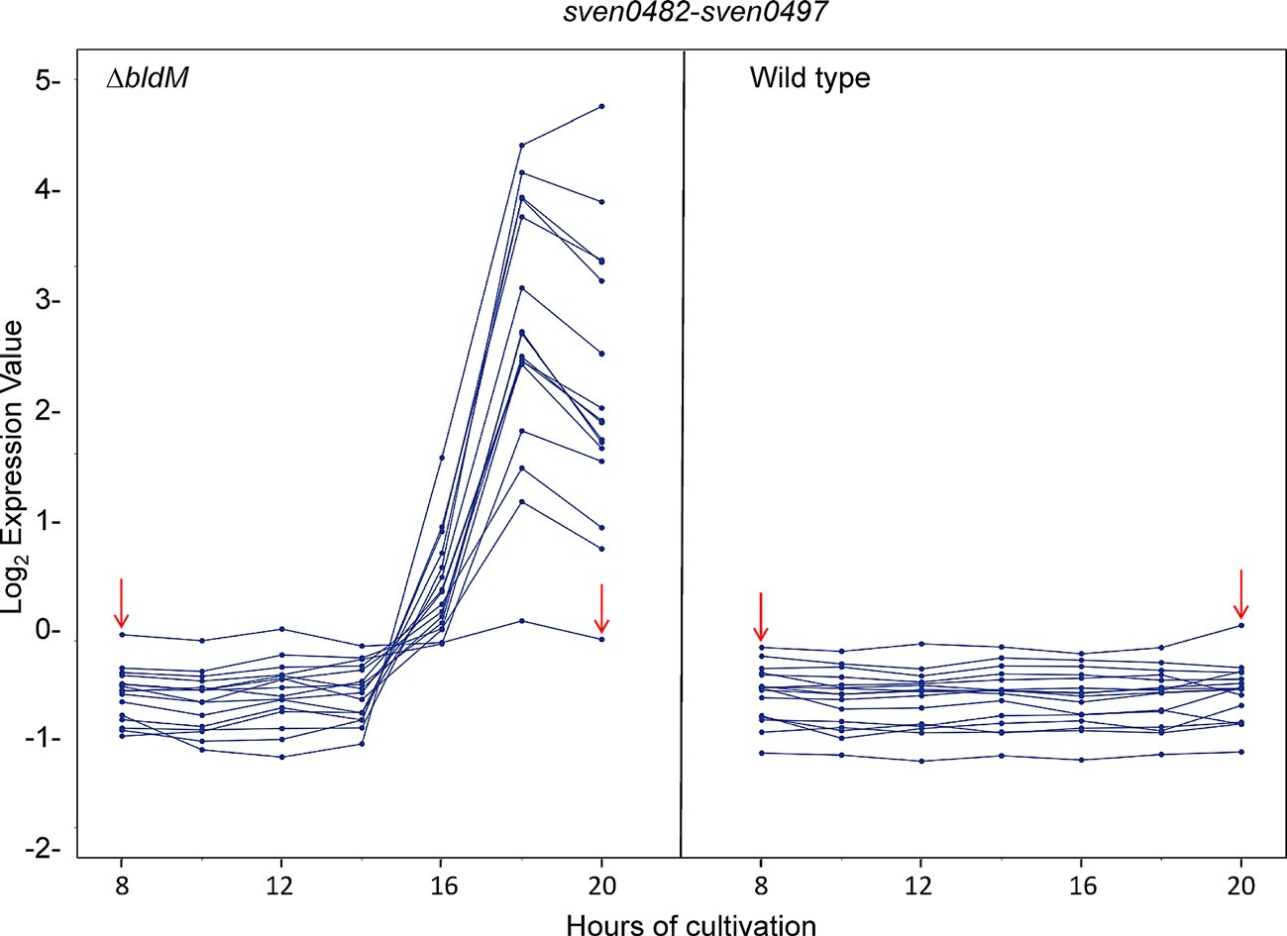
Microarray expression profiles of *sven0482–sven0497* in the *S. venezuelae bldM* mutant (left) in comparison with the wild‐type strain (right); the profiles for *sven0484* are indicated by red arrows. The *y*‐axis represents normalised transcript abundance and the *x*‐axis indicates the times, in hours, after which cultures were harvested for analysis.

**Scheme 1:**
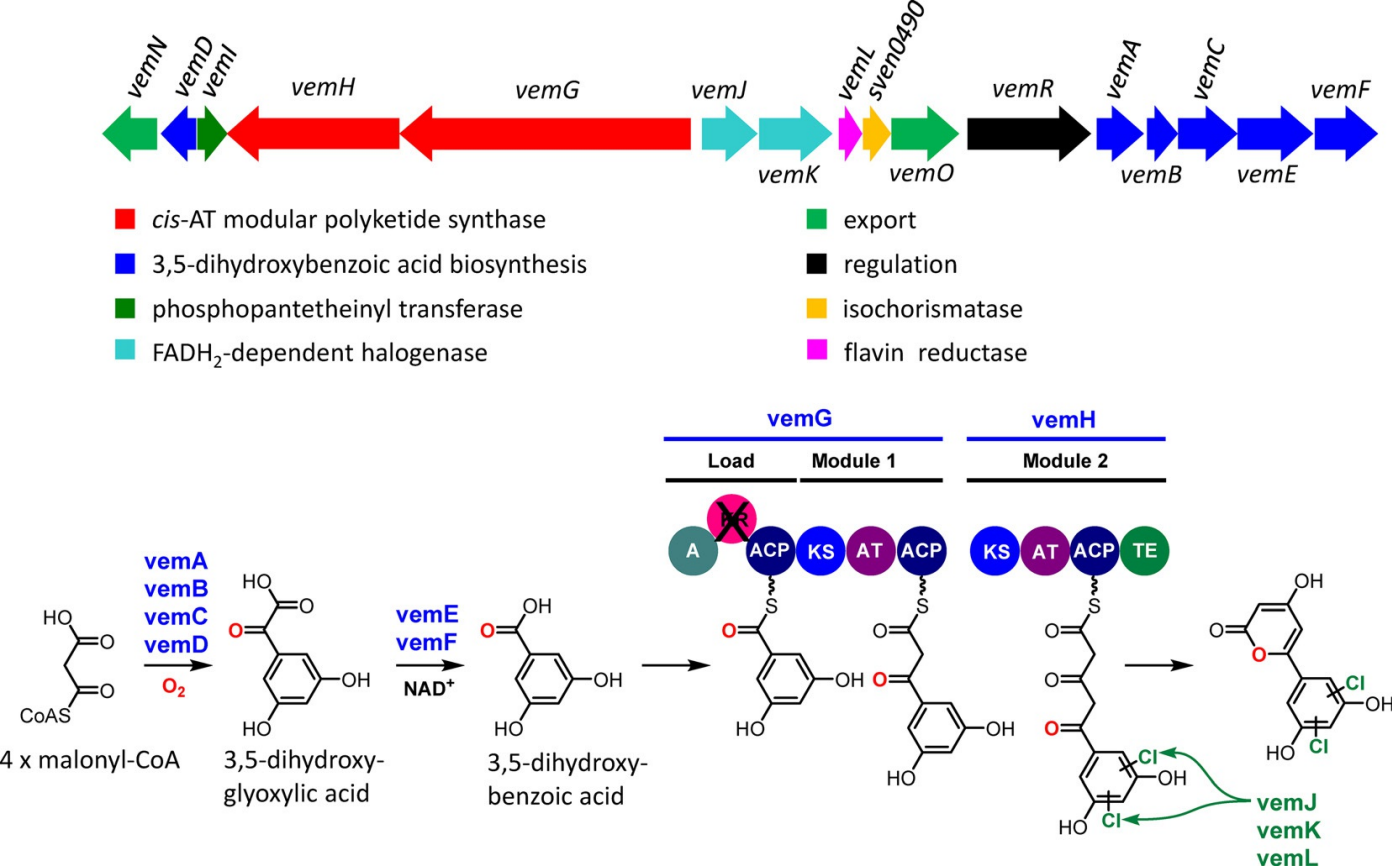
Organisation of the *vem* cluster in *S. venezuelae* and proposed pathway for the biosynthesis of venemycin. The presence of two genes (*vemJ*, *vemK*) encoding FAD‐dependent halogenases suggests that halogenated derivatives of venemycin might also be produced (the examples given are of chlorinated derivatives).

Comparative microarray analysis of wild‐type *S. venezuelae* and a congenic *bldM* mutant (strain SV13)^18^ revealed the presence of a previously unidentified cryptic polyketide biosynthetic gene cluster (*vem*), expression of which was markedly upregulated in the mutant (Figure 1). The cluster is approximately 29.5 kb in size and contains 15 upregulated genes: *sven0482*, *sven0483*, and *sven0485–sven0497*. The transcriptional profiles of the flanking genes *sven0477–sven0481* and *sven0498–sven0502*, which were expressed at low levels both in the wild‐type strain and in the *bldM* mutant (data not shown), indicated that they were unlikely to be functionally related to the *vem* cluster (*sven0482*–*sven0497*). From the amino acid sequence similarity of the encoded proteins to those present in the NCBI protein database we predicted their putative functions (Table 1) and proposed a biosynthetic pathway (Scheme 1) that, if correct, would result in the production of a new biaryl metabolite—which we named venemycin—and halogenated derivatives.

**Table 1:** Proposed functions of genes within the venemycin biosynthetic gene cluster.

Gene, *sven* number (number of encoded aas)	Proposed function	Closest homologue with known/predicted function (in NCBI database 27/06/16)	Identity [%] (identity/length of alignment)
*vemA*, *sven0493* (361)	(3,5‐dihydroxyphenyl)acetyl‐CoA biosynthesis	CAQ52620; type III PKS [*S. violaceoruber*]	71 (253/356)
		CAE53371; DpgA protein [*Actinoplanes teichomyceticus*]	67 (233/346)
*vemB*, *sven0494* (236)	(3,5‐dihydroxyphenyl)acetyl‐CoA biosynthesis	CAC48379; DpgB, enoyl‐CoA hydratase [*Amycolatopsis balhimycina* DSM 5908]	50 (106/211)
*vemC*, *sven0495* (462)	conversion of (3,5‐dihydroxyphenyl)acetyl‐CoA into (3,5‐ dihydroxyphenyl)glyoxylic acid	AHF20612; dioxygenase [*Amycolatopsis* sp. MJM2582]	61 (263/434)
		CAQ52618; dioxygenase [*S. violaceoruber*]	56 (250/447)
*vemD*, *sven0483* (267)	(3,5‐dihydroxyphenyl)acetyl‐CoA biosynthesis	WP_023546756; enoyl‐CoA hydratase [*Streptomyces roseochromogenus*]	85 (228/267)
		CAE53374; DpgD, enoyl‐CoA hydratase [*A. teichomyceticus*]	67 (175/206)
*vemE*, *sven0496* (577)	TDP‐dependent decarboxylase responsible for conversion of (3,5‐dihydroxyphenyl)glyoxylic acid into 3,5‐dihydroxy‐ benzaldehyde	WP_023546769; thiamine‐pyrophosphate‐binding protein [*Streptomyces roseochromogenes*]	86 (497/575)
		CAQ52617; benzoylformate decarboxylase [*S. violaceoruber*]	65 (378/580)
*vemF*, *sven0497* (486)	aldehyde dehydrogenase responsible for conversion of 3,5‐ dihydroxybenzaldehyde into 3,5‐dihydroxybenzoic acid	WP_023546770; benzaldehyde dehydrogenase [*S. roseochromogenus*]	87 (419/483)
		CAQ52616; benzaldehyde dehydrogenase [*S. violaceoruber*]	67 (322/480)
*vemG*, *sven0486* (2236)	type I modular PKS	WP_053663290; type I polyketide synthase [*Streptomyces* sp. NRRL F‐7442]	72 (1646/2289)
*vemH*, *sven0485* (1325)	type I modular PKS	WP_023546758; type I polyketide synthase [*S. roseochromogenus*]	80 (1069/1333)
		AEZ54379; PieA6, polyketide synthase [*Streptomyces piomogenus*]	52 (699/1338)
*vemI*, *sven0484* (231)	4‐phosphopantetheinyl transferase	WP_051820474; 4‐phosphopantetheinyl transferase [*Streptomyces flavochromogenes*]	72 (165/228)
*vemJ*, *sven0487* (425)	FADH_2_‐dependent halogenase	EST32607; tryptophan halogenase [*S. roseochromogenus* ssp. *oscitans* DS 12.976]	94 (400/425)
*vemK*, *sven0488* (561)	FADH_2_‐dependent halogenase	WP_023546761; FAD‐dependent oxidoreductase [*S. roseochromogenus*]	93 (521/559)
*vemL*, *sven0489* (175)	flavin reductase	WP_023546762; oxidase [*S. roseochromogenus*]	86 (151/175)
*sven0490* (216)	isochorismatase	WP_030556570; isochorismatase [*Streptomyces exfoliatus*]	52 (115/222)
*vemN*, *sven0482* (451)	MPS transporter	WP_055468166; MPS transporter [*Streptomyces* sp. NBRC 110030]	76 (343/451)
*vemO*, *sven0491* (520)	transporter	WP_050486995; MFS transporter [*Streptomyces* sp. CNS654]	87 (439/503)
*vemR*, *sven0492* (949)	LAL pathway‐specific activator	GAT71280; ATPase [*Planomonospora sphaerica*]	39 (372/946)

We propose that the biosynthesis of venemycin begins with the formation of 3,5‐dihydroxybenzoic acid catalysed by the enzymes encoded by *vemA*, *vemB*, *vemC*, *vemD*, *vemE* and *vemF*. VemA–F are homologues of Ken2–7, which have been proposed to catalyse the formation of the 3,5‐dihydroxybenzoic acid starter unit utilised by a type I modular PKS for the assembly of kendomycin in *Streptomyces violaceoruber*.^20^ VemA has 60–70 % identity to DpgA, a type III PKS that has been shown to catalyse the conversion of four malonyl‐CoA molecules to (3,5‐dihydroxyphenyl)acetyl‐CoA.^21^ VemB and VemD are homologues of DpgB and DpgD, respectively, which have been shown to increase the efficiency of the DpgA‐catalysed reaction.^21^ VemC is a homologue of DpgC, a cofactor‐independent dioxygenase that has been shown to catalyse the conversion of (3,5‐dihydroxyphenyl)acetyl‐CoA to (3,5‐dihydroxyphenyl)oxoacetic acid.^22^ The (3,5‐dihydroxyphenyl)glyoxylic acid produced by the DpgA–D enzymes, which are universally conserved in glycopeptide‐producing Actinobacteria, is converted into (3,5‐dihydroxyphenyl)glycine, a nonproteinogenic amino acid incorporated into antibiotics such as balhimycin,^23^ vancomycin,^21^ teicoplanin^24^ and A47934.^25^, ^26^ It is proposed that VemE catalyses the thiamine‐diphosphate (TDP)‐dependent decarboxylation of (3,5‐dihydroxyphenyl)oxoacetic acid to form 3,5‐dihydroxybenzaldehyde, which is oxidised to 3,5‐dihydroxybenzoic acid by VemF, a putative nicotinamide‐dependent dehydrogenase.

We suggest that 3,5‐dihydroxybenzoic acid serves as a starter unit for the assembly of venemycin by the type I modular PKSs VemG and VemH. Conserved domain searches revealed that VemG contains at its N terminus a loading module consisting of an adenylation (A) domain, a ketoreductase (KR) domain (predicted to be nonfunctional because it lacks a canonical YXXXN motif at its active site, even though it appears to contain an NADPH binding site) and an ACP domain. The A domain has 63 % amino acid sequence similarity to the corresponding domain at the N terminus of the type I modular PKS responsible for the assembly of kendomycin, which has been proposed to load a 3,5‐dihydroxybenzoic acid starter unit onto the PKS.^20^ At its C terminus, VemG has a chain elongation module that contains KS, AT and ACP domains. VemH is predicted to contain a second chain elongation module consisting of KS, AT and ACP domains, followed by a thioesterase (TE) domain. The AT domains in VemG and VemH are both predicted to be specific for malonyl‐CoA.^27^ The TE domain likely catalyses release of the fully assembled polyketide chain to form the pyrone, although hydrolytic release to form a somewhat less stable 3,5‐diketo acid would also be possible. VemI is a phosphopantetheinyl transferase (PPTase) that is proposed to be responsible for posttranslational phosphopantetheinylation of the three ACP domains within the modular PKS; surprisingly, because it is embedded in the cluster, transcription of *vemI* (*sven0484*) was not markedly increased in the *bldM* mutant (Figure 1), and it is conceivable that under the growth conditions used this function is mainly performed by one of the other PPTases encoded in the *S. venezuelae* genome by *sven0914*, *sven6190*, *sven6269* and *sven4419* (the last of these is likely to be the PPTase used in fatty acid synthesis). Either of the aromatic rings of venemycin could then be halogenated by one or both of the two putative flavin‐dependent halogenases, VemJ and VemK, with the FADH_2_ cofactor required by these enzymes being supplied by the flavin reductase VemL.

VemN and VemO belong to the major facilitator superfamily (MFS) transporters and are presumably involved in the export of venemycin. VemR is a member of the large ATP‐binding LuxR‐like (LAL) family of transcriptional regulators and is proposed to function as a pathway‐specific activator of the biosynthetic gene cluster. The protein contains a N‐terminal AAA‐ATPase domain and a C‐terminal LuxR family DNA‐binding domain with a helix‐turn‐helix motif. LAL homologues have been shown to activate the production of several actinomycete specialised metabolites, including pikromycin (PikD),^28^ rapamycin (RapH)^29^ and the stambomycins (SAMR0484).^30^ Sven0490 is homologous to isochorismatases that convert isochorismate into 2,3‐dihydro‐2,3‐dihydroxybenzoate. These enzymes commonly participate in the biosynthesis of nonribosomal peptide siderophores, and there is no apparent role for Sven0490 in venemycin biosynthesis.

### Identification of venemycin and a chlorinated derivative

Our proposed biosynthetic pathway (Scheme 1) predicted that *S. venezuelae* should produce a potentially new metabolite with the molecular formula C_11_H_8_O_5_, as well as halogenated derivatives. On the basis of this prediction, we set out to identify and characterise the products of the *vem* gene cluster by comparative metabolite profiling of culture supernatants from the *S. venezuelae* Δ*bldM* mutant (strain SV13) and its parental strain after growth for four days in liquid maltose‐yeast extract‐malt extract medium made with tap water (MYM‐TAP) and supplemented with trace elements. LC‐HRMS identified molecular ions corresponding to [*M*+H]^+^ for venemycin [*m/z* calcd for C_11_H_9_O_5_^+^: 221.0444; found: 221.0443 (an absorbance maximum at 324 nm was observed in the UV/visible spectrum for this species; Figure S1 in the Supporting Information)] and a monochlorinated derivative [*m/z* calcd for C_11_H_8_O_5_Cl^+^: 255.0055; found: 255.0051 (Figure S2)] that were present only in the culture supernatant of the mutant strain. The compounds were detectable at 48 h of growth and their levels peaked between 72 and 96 h; after 120 h they had declined by 30 % (data not shown).

### Heterologous expression of the *vem* gene cluster in *S. coelicolor* and structure elucidation of venemycin

Because the levels of production of venemycin and its monochlorinated derivative by the *S. venezuelae* Δ*bldM* mutant were insufficient to permit further structural analysis, and to confirm that the *vem* gene cluster does indeed direct venemycin biosynthesis, we set out to express the gene cluster in a heterologous host. The cosmid derivative pIJ13035 (Table S1) containing the entire *vem* gene cluster was introduced into *Streptomyces coelicolor* M1152^31^ and M1316^32^ (a derivative of M1152 from which two native type III PKS genes have been deleted; Table S1) by conjugation from *Escherichia coli*. Chromosomal integration was confirmed by PCR analysis, yielding *S. coelicolor* M1818 and M1819, respectively. The strains were grown as described above, and their culture supernatants were analysed by LC‐MS. Neither venemycin nor its chlorinated derivative was detected (data not shown); this probably reflects a lack of transcription of the *vem* gene cluster in the heterologous hosts.

**Figure 2:**
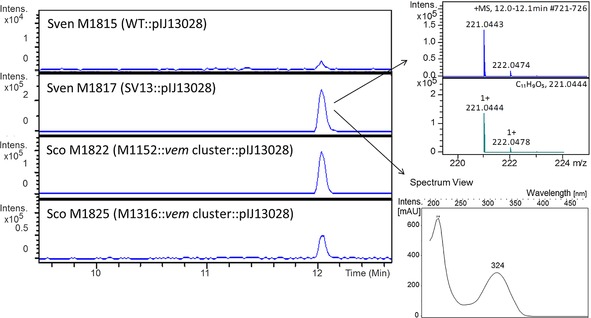
Left: Extracted ion chromatograms at *m*/*z* 221.04±0.1 from positive‐ion‐mode UHPLC‐ESI‐TOF‐MS analyses of culture supernatants from strains containing pIJ13028 (ermE*p::*vemR*). M1815, *S. venezuelae* wild‐type; M1817, *S. venezuelae* (Δ*bldM*); M1822, *S. coelicolor* M1152 containing the *vem* gene cluster; M1825, *S. coelicolor* M1316 containing the *vem* gene cluster. The strains were grown in MYM‐TAP medium for four days before sampling. Right: Mass and UV/Vis spectra for the analyte with a retention time of ≈12 min. Comparison of the measured mass spectrum with the simulated spectrum for C_11_H_9_0_5_^+^ established the molecular formula of venemycin as C_11_H_8_O_5_.

**Scheme 2:**
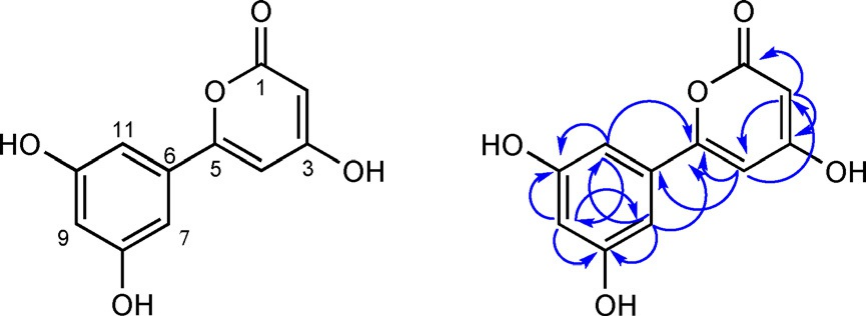
Structure of venemycin (left) and correlations observed in its HMBC spectrum (right).

**Figure 3:**
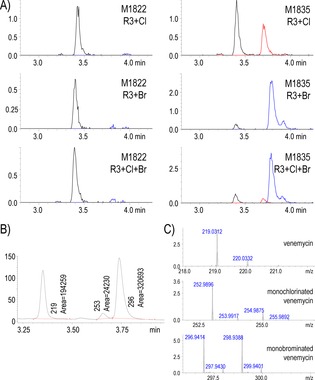
LC‐MS analyses (negative‐ion mode) of venemycin and halogen‐ ated derivatives produced by *S. coelicolor* M1822 (M1152+*vem* gene cluster+ermE*p::*vemR*) and *S. coelicolor* M1835 (M1822+ermE*p::*vemJKL*) grown for seven days in R3 medium supplemented with NaCl alone, NaBr alone or NaCl+NaBr. A) Extracted ion chromatograms for *m*/*z* 219.0299 (**—**), 252.9909 (**—**) and 296.9404 (**—**), corresponding to calculated [*M*−H]^−^ *m*/*z* for venemycin and its monochlorinated and monobrominated derivatives, respectively (*y*‐axis, intensity units ×10^6^). B) Extracted UV/visible chromatogram at 329 nm (maximum of absorbance under these conditions; *y*‐axis=mAU) for sample “M1835+Cl+Br”, illustrating the ratio of conversion of venemycin to its singly chlorinated or brominated derivatives when the three halogenation genes are constitutively expressed. C) MS spectra (zoomed‐in) of the peaks corresponding to each of the venemycin species (*y*‐axis, intensity units×10^6^).

To surmount this problem, *vemR*, encoding a putative transcriptional activator of the LAL family, was cloned downstream of the constitutive ermE* promoter within the integrative pIJ10257 vector,^33^ and the resulting plasmid pIJ13028 was introduced into both *S. coelicolo*r M1818 and M1819 by conjugation from *E. coli*, yielding *S. coelicolor* M1822 (M1152+*vem* cluster+ermE*p::*vemR*) and *S. coelicolor* M1825 (M1316+*vem* cluster+ermE*p::*vemR*), respectively. Chromosomal integration of pIJ13028 in each of the recipient strains was confirmed by PCR analysis (data not shown). Cultivation of both M1822 and M1825 resulted in the production of a metabolite with the same molecular formula, retention time and MS/MS fragmentation pattern as venemycin produced by *S. venezuelae* SV13 (Figure 2). Sometimes, but not always, a very small amount of a singly chlorinated derivative of venemycin was also detected in the culture supernatants of these strains (data not shown). *S. coelicolor* derivatives containing pIJ13028 but lacking the *vem* gene cluster did not produce venemycin or its chlorinated derivative (data not shown). This confirmed that pIJ13035 contains all of the genes required for venemycin biosynthesis. Introduction of pIJ13028 into wild‐type *S. venezuelae* (yielding M1815) also resulted in venemycin production (Figure 2), thus providing further evidence that VemR is a transcriptional activator. Levels of production were further increased when the same plasmid was introduced into the *S. venezuelae* Δ*bldM* mutant (to give M1817), yielding sufficient venemycin for structure elucidation by NMR spectroscopic analysis (Scheme 2 and Table S3). Venemycin was purified from M1817 by semipreparative HPLC and analysed by ^1^H and HSQC and HMBC NMR experiments (Figures 3 and S3–S6). The NMR analyses established that 3,5‐dihydroxybenzoic acid had co‐purified with venemycin, consistent with its proposed role as an intermediate in venemycin biosynthesis (Figures S3–S7). A search of the SciFinder database established that although venemycin had not been reported previously as a natural product it is a known synthetic compound with potent superoxide anion radical scavenging activities.^34^

### Halogenation of venemycin

Because production levels of the monochlorinated derivative of venemycin were low and no doubly halogenated venemycin derivatives could be detected despite the presence of two genes (*vemJ* and *vemK*) encoding putative FADH_2_‐dependent halogenases, we decided to express *vemJ*, *vemK* and *vemL* (encoding a flavin reductase) from the strong constitutive ermE* promoter. The genes were cloned into the conjugative multicopy vector pIJ12477^35^ (Table S1), and the resulting plasmid—pIJ13029 (ermE*p::*vemJKL*)—was introduced into *S. venezuelae* M1815 (wild‐type+ermE*p::*vemR*) and M1817 (Δ*bldM*+ ermE*p::*vemR*), as well as *S. coelicolor* M1822 (M1152+*vem* cluster+ermE*p::*vemR*) and M1825 (M1316+*vem* cluster+ ermE*p::*vemR*), yielding strains M1827, M1831, M1835 and M1839, respectively. The strains were grown as before in liquid MYM‐TAP medium supplemented with trace elements, and the culture supernatants were analysed by LC‐HRMS in negative‐ion mode, which resulted in increased sensitivity relative to the earlier analyses carried out in positive‐ion mode. Venemycin and its monochlorinated derivative were detected in all of the cultures (data not shown), but the amounts produced were still insufficient to permit further structural characterisation of the chlorinated form, and no polyhalogenated venemycin derivatives were detected. From HPLC profiles with UV‐ and visible‐range detection, the levels of production of venemycin and the monochlorinated form in *S. coelicolor* strains M1835 and M1839 were generally about half those observed in the *S. venezuelae* Δ*bldM* derivative M1817 (data not shown). The two heterologous hosts produced similar amounts.

Some flavin‐dependent halogenases prefer bromide over chloride,^36^ but because chloride is the most abundant halide in commonly used culture media, it is chlorine rather than bromine that is usually incorporated into metabolic products under laboratory conditions. To test whether bromide ions are accepted or even preferred by VemJ/VemK, *S. venezuelae* M1831 (Δ*bldM*+ermE*p::*vemR*+ermE*p::*vemJKL*) and *S. coelicolor* M1835 (M1152+*vem*‐cluster+ermE*p::*vemR*+ ermE*p::*vemJKL*) were cultivated in a minimal medium (MM) supplemented with NaCl or NaBr. A monobrominated venemycin derivative was readily detected in the culture supernatants of both strains (Figure S8). Because heterologous expression in *S. coelicolor* had worked well (Figures 3 and S8), we repeated the experiment with M1822 and M1835 in modified R3 medium supplemented with NaCl, NaBr, or equimolar amounts of both. A 13:1 ratio of monobrominated to monochlorinated venemycin derivatives was observed in the culture containing equimolar amounts of NaCl and NaBr, thus indicating that bromide is the preferred substrate for one or both of the halogenases encoded in the venemycin biosynthetic gene cluster (Figure 3). Although the monobrominated venemycin derivative constituted almost 60 % of the venemycin‐related metabolites produced in the culture containing NaBr, the amount produced was not sufficient to permit further structural analysis.

Halogenation of a natural product can significantly affect its biological activity, including its antibacterial activity.^37^, ^38^, ^39^ We thus investigated the activity of culture supernatants of M1835 containing venemycin, and its monochlorinated and monobrominated derivatives, against *Micrococcus luteus* and *E. coli*, but no growth inhibition was observed (data not shown).

### Transcriptional organisation of the *vem* gene cluster

**Figure 4:**
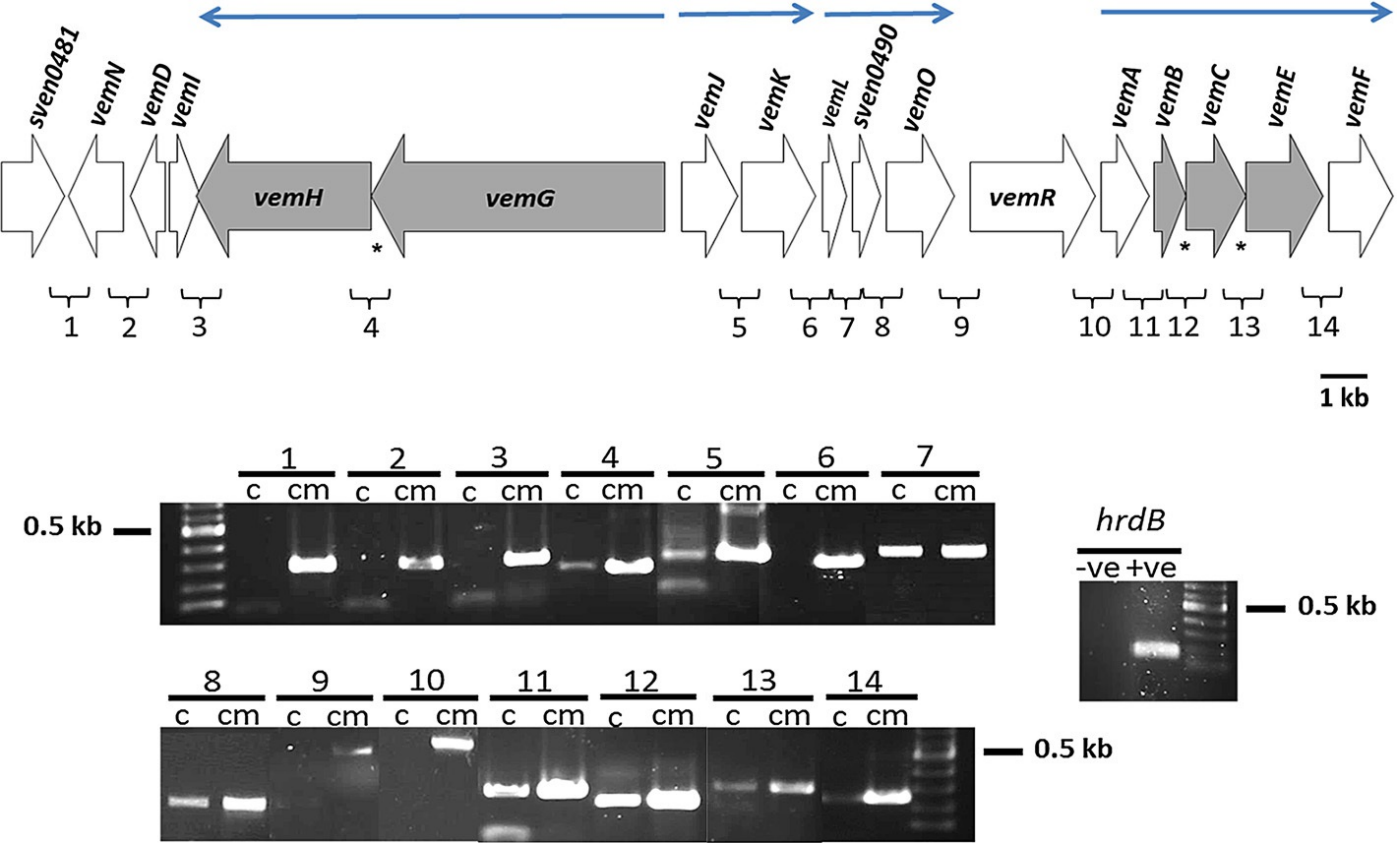
RT‐PCR was used to identify likely operons (**→**) in the *vem* cluster. Translationally coupled genes are shaded in grey, and asterisks indicate occurrences of translational coupling. “c” and “cm” indicate amplification from cDNA and cosmid DNA, respectively. *hrdB* of *S. coelicolor* M145 was used as an internal control. *hrdB* (−ve) indicates attempted PCR amplification of *hrdB* from RNA extracted from *S. coelicolor* M1825 without cDNA synthesis. *hrdB* (+ve) indicates PCR amplification of *hrdB* from *S. coelicolor* M145 genomic DNA. A 100 bp DNA ladder (NEB) was used as the DNA size marker.

Inspection of the sequence of the *vem* gene cluster (Figure 1) revealed the presence of multiple transcription units, and several potential operons. To throw light on how expression of the gene cluster might be regulated, and to provide a basis for future attempts to increase the level of productivity—by engineering enhanced levels of transcription, for example—the operon structure of the *vem* gene cluster was determined. RNA was isolated from mycelium of *S. coelicolor* M1825 (M1316+*vem* cluster+ermE*p::*vemR*) after two days of incubation, corresponding to the onset of venemycin production, and cDNA was synthesised by a two‐step RT‐PCR protocol with random hexamers in the presence of reverse transcriptase (RT). The resulting cDNA was used as a template for PCR amplification with primers flanking 14 intergenic regions within the proposed *vem* gene cluster (Table S2). The fidelity of the primers used was confirmed by using pIJ13035 DNA as template. Each pair produced a single PCR product of the predicted size. The results suggested the existence of eight transcription units: *vemN*, *vemD*, *vemI*, *vemGH*, *vemJK*, *vemL‐sven0490‐vemO*, *vemR* and *vemABCEF* (Figure 4). Attempts to use RT‐PCR to amplify cDNA derived from *hrdB*, encoding the housekeeping *σ* factor of *S. coelicolor*, by using RNA treated with and without reverse transcriptase and 35 cycles of amplification confirmed the absence of genomic DNA.

## Conclusion

By combining microarray analysis of a *S. venezuelae* Δ*bldM* mutant with bioinformatics analysis of its genome sequence, we were able to identify a biosynthetic gene cluster encoding an unusual combination of modular type I and type III PKSs that was predicted to assemble a new biaryl natural product—which we named venemycin—along with halogenated derivatives. Comparative analysis of the metabolites produced by the wild‐type strain and the Δ*bldM* mutant by using UHPLC‐ESI‐Q‐TOF‐MS identified two compounds with molecular formulae corresponding to venemycin and a monochlorinated derivative. However, the quantities of these metabolites were insufficient to permit structure elucidation by NMR spectroscopic analysis. Thus, *vemR*, which encodes a putative LAL transcriptional activator, was constitutively expressed in *S*. v*enezuelae* and in *S. coelicolor* derivatives containing the cloned *vem* gene cluster. This provided sufficient quantities of venemycin for NMR analysis, thus confirming that it has the structure predicted by bioinformatics. Although the *vem* gene cluster contains two halogenase genes (*vemJ* and *vemK*), we were only able to detect the production of monohalogenated venemycin derivatives. It is conceivable that one of the halogenases is not functional, or that each of the halogenases modifies venemycin at a different position and the monohalogenated venemycin derivatives are not substrates for the remaining halogenase. In a competitive feeding experiment employing equimolar concentrations of chloride and bromide, the monobrominated venemycin derivative(s) was/were produced at a 13‐fold higher level than the monochlorinated derivative(s), thus indicating that one or both of the halogenases has a significant preference for the larger halide ion. Although venemycin is produced in part by a type III PKS, there were no significant differences in the levels of production in M1152 and M1316, a derivative of M1152 in which two type III PKS genes have been deleted and in which precursor supply for heterologously synthesised venemycin might have been better. It remains to be seen whether this is true for other metabolites derived from malonyl‐CoA. Our biosynthetic scheme has no role for *sven0490* (encoding a putative isochorismatase), and indeed, deletion of this gene in *S. coelicolor* M1825 had no effect on venemycin production (A. Thanapipatsiri, unpublished data). This is particularly surprising because *sven0490* is co‐transcribed with *vemL* and *vemO*.

## Experimental Section

**Bacterial strains, plasmids, cosmids and culture conditions**: The strains, plasmids and cosmids used in this study are listed in Table S1. *E. coli* strains were grown and manipulated by standard methods.^40^, ^41^, ^42^ For generating spore stocks, *S. venezuelae* and *S. coelicolor* were grown on MYM‐TAP agar^43^ and mannitol soya flour (SFM) agar,^44^ respectively. MYM‐TAP liquid medium (with 400 μL of trace element solution^44^ added per 200 mL of medium added after autoclaving) was used to grow *Streptomyces* strains for venemycin production and for RNA isolation from *S. coelicolor* M1825. For the halogenation studies we used Minimal Medium (MM)^44^ supplemented with NaCl or NaBr (0.5 g L^−1^), and modified versions of R3 medium^45^ consisting of glucose (10 g L^−1^), yeast extract (5 g L^−1^), casamino acids (100 mg L^−1^), proline (3 g L^−1^), MgSO_4_ **⋅**7 H_2_O (10 g L^−1^), CaSO_4_ **⋅**2 H_2_O (4 g L^−1^), K_2_SO_4_ (200 mg L^−1^), KH_2_PO_4_ (50 mg L^−1^), TES [*N*‐tris(hydroxymethyl)methyl‐2‐aminoethanesulfonic acid, 5.6 g L^−1^], trace elements (as described for R2YE medium^44^) and NaCl or NaBr or both (1 g L^−1^); the media were adjusted to pH 7.2 with NaOH and autoclaved. *Streptomyces* liquid cultures were performed in medium (50 mL) in 250 mL flasks containing stainless steel springs to promote dispersed growth with at least two replicates. The cultures were grown at 30 °C with shaking at 250 rpm for up to five days. Antibiotics were added to the media at the following final concentrations when appropriate: apramycin (50 μg mL^−1^), nalidixic acid (25 μg mL^−1^), kanamycin (10 μg mL^−1^ for *S. venezuelae* and 50 μg mL^−1^ for *S. coelicolor*), and hygromycin (25 μg mL^−1^ for *S. venezuelae* and 50 μg mL^−1^ for *S. coelicolor*).

**Microarray analysis**: RNA isolation from *S. venezuelae* and subsequent DNA microarray analysis were reported previously,^46^ and the data were deposited in the arrayExpress database (https://www.ebi.ac.uk/arrayexpress/) under accession number E‐MTAB‐2716. Briefly, data in CEL files produced by the chip scanner were normalised by using robust multi‐array average (RMA) as implemented in the Affy package of Bioconductor (https://www.bioconductor.org/). Replicates were combined by using the lmFit and eBayes functions of the limma package of Bioconductor. This resulted in a table of log 2 expression values for each gene at various time points in the different strains. For the analysis reported here, the log 2 expression values for the relevant genes (*sven0477–sven0481, sven0482–sven0497* and *sven0498–sven0502*) in the *bldM* mutant and the wild‐type strains were extracted from the genome‐wide data set, centred to a mean of zero for each gene (by using the scale function of R) and then plotted by using R (https://www.R‐project.org/).

**DNA and RNA manipulations**: DNA extraction from *E. coli* was carried out by standard methods^42^ or with a QIAprep Spin Miniprep Kit (Qiagen). Restriction enzymes (Roche and NEB) were used according to the instructions provided by the manufacturers. PCR amplifications were performed with Taq DNA polymerase (Qiagen). The amplification of DNA fragments used in the expression constructs was performed with Phusion High‐Fidelity DNA Polymerase (NEB). Extraction of RNA from *Streptomyces* strains was performed with a RNeasy mini kit (Qiagen, Supporting Information). RT‐PCR was performed with a Maxima First Strand cDNA Synthesis kit (Thermo Scientific). Amplification of DNA from synthesised cDNA was carried out with Taq DNA polymerase (Qiagen).

**Annotation of the** ***vem*** **cluster**: The *Streptomyces* genome server (StrepDB: http://strepdb.streptomyces.org.uk) was used to derive the amino acid sequences for each of the proteins encoded by the *vem* cluster. These were then used in BlastP searches of the NCBI protein database to assign the probable function of each gene.

**Generation of a transmissible cosmid for heterologous expression of the** ***vem*** **cluster in** ***S. coelicolor***: PCR targeting^41^ of cosmid SV3E02, which contains the *vem* cluster, was carried out to replace *neo* of the SuperCos1 vector with a 5247 bp *oriT‐attP‐int‐aac(3)IV* fragment (flanked by SspI recognition sites), which was isolated by SspI restriction digestion of pMJCOS1^47^ (now known as pIJ10702, Figure S9). Replacement occurred through double‐crossover recombination at homologous flanking sequences to yield the conjugative and integrative pIJ13035, which was then introduced into the heterologous hosts *S. coelicolor* M1152 and *S. coelicolor* M1316 (Table S1) by intergeneric conjugation,^44^ whereupon it integrated into the host's genome at the chromosomal φC31 *attB* site to yield *S. coelicolor* M1818 and *S. coelicolor* M1819, respectively. The ex‐conjugants were selected on SFM agar containing apramycin and nalidixic acid and streaked for a couple of passages on the same agar to ensure complete removal of the *E. coli* donor strain. Chromosomal integration of pIJ13035 was confirmed by PCR amplification of selected *vem* genes by using two primer pairs: SVEN0487TF/SVEN0487TR (flanking *vemJ*) and SVEN0493TF/SVEN0493TR (flanking *vemA*, Table S2). The resulting *S. coelicolor* strains were grown until fully sporulated, and the spores were harvested and stored at −20 °C in glycerol (20 %).^44^

**Constitutive expression of** ***vemR***: The *vemR* coding sequence (from start to stop codon) was amplified from cosmid SV3E02 by using primers SVEN0492NdeIF1 and SVEN0492HindIIIR (Table S2) and blunt‐end cloned into pBlueScript II KS(+) at the SmaI site. Restriction enzyme digestion and DNA sequencing by using M13 universal primers and designed sequencing primers SVEN0492NW1, SVEN0492NW2, SVEN0492W3, SVEN0492W4 and SVEN0492NW5 (Table S2) were used to verify the cloned fragment, which was then inserted downstream of the constitutive ermE* promoter in pIJ10257 (which integrates at the host's chromosomal φBT *attB* site^33^), creating pIJ13028 in *E. coli* DH5α (Figure S10).^42^ pIJ13028 and pIJ10257 were then introduced by conjugation into *S. venezuelae* wild type, *S. venezuelae* SV13 (Δ*bldM*), *S. coelicolor* M1818 (M1152::pIJ13035) and *S. coelicolor* M1819 (M1316::pIJ13035). pIJ13028 was also introduced into *S. coelicolor* M1152 and *S. coelicolor* M1316 to generate *vemR* expression control strains. *S. coelicolor* ex‐conjugants were selected on SFM agar containing hygromycin and nalidixic acid whereas MYM‐TAP agar was used for the *S. venezuelae* strains. The resulting strains were streaked on the same media containing hygromycin and nalidixic acid until the strains were free of *E. coli* cells. The resulting recombinant strains were verified by PCR with primers pIJ86F1 and SVEN0492HindIIIR (Table S2) and grown until fully sporulated, and the spores were harvested and stored at −20 °C in glycerol (20 %).

**Constitutive expression of halogenase genes**: A PCR fragment containing the coding sequences of *vemJKL* and the ribosomal binding site of *vemJ* was amplified by using primers SVEN0487BamHIF and SVEN0489HindIIR and cloned into pBluescript II KS(+) cleaved with SmaI. The cloned fragment was verified by restriction enzyme digestion and DNA sequencing with use of M13 universal primers and designed sequencing primers SVEN0487W1, SVEN0488W1, SVEN0488W2 and SVEN0489W1 (Table S2) and inserted downstream of the constitutive ermE* promoter in the multicopy pIJ12477 cut with BamHI and HindIII, creating pIJ13029 (ermE*p::*vemJKL*, Figure S10). pIJ13029 and pIJ12477 (vector‐only control) were then introduced into *Streptomyces* strains by intergeneric conjugation as previously described.^44^ *S. coelicolor* ex‐conjugants were selected on SFM agar containing kanamycin and nalidixic acid, whereas MYM‐TAP agar containing trace element solution and kanamycin was used for the *S. venezuelae* ex‐conjugants. Ex‐conjugants were verified by PCR amplification with primer pair pIJ86F1 and pIJ86R2 (Table S2), which anneal at sites within pIJ12477 220 and 229 bp from the BamHI and HindIII cloning sites of the vector, respectively. The resulting strains were grown on agar, and spores were stored as previously described.

**Analysis of culture supernatants**: Stationary‐phase cultures were centrifuged at 13 000 rpm in a fixed angle rotor refrigerated microcentrifuge at 4 °C for 20 min to remove mycelium. Samples were analysed by using two different systems and sets of conditions:

*A) A Shimadzu LC‐MS system equipped with a NexeraX2 liquid chromatograph (LC30AD) fitted with a Prominence photodiode array detector (SPD‐M20A) and an LC‐MS‐IT‐ToF mass spectrometer*: Samples (typically 5 μL) were injected onto a Kinetex XB C_18_ (2.6 μm, 100 Å, 50 mm by 2.1 mm) column (part no. 00B‐4496‐AN, Phenomenex, USA) fitted with a KrudKatcher Ultra HPLC inline filter (part no. AF0—8497, Phenomenex), kept at 40 °C and eluted at a flow rate of 0.6 mL min^−1^ with a gradient of 0.1 % formic acid in water and methanol, from 2 % to 100 % methanol over 7 min. Data acquisition and analysis were performed with LCMS solution version 3 (Shimadzu).

*B) A Dionex 3000RS UHPLC coupled to a Bruker MaXis Q‐TOF mass spectrometer with an Agilent Zorbax Eclipse plus column (C18, 100×2.1 mm, 1.8 μm)*: Mobile phases consisted of A (water with 0.1 % formic acid) and B (acetonitrile with 0.1 % formic acid). A gradient of 5 % B to 100 % B in 15 min was employed with a flow rate of 0.2 mL min^−1^, with UV absorption set at 254 nm.

**Purification of venemycin by semipreparative HPLC and NMR spectroscopy**: Venemycin was purified from the concentrated culture supernatant by semipreparative HPLC on a reversed‐phase column (C_18_, 100×21 mm, fitted with a C_18_ pre‐column 10×21 mm). The mobile phase contained water and acetonitrile, both with formic acid (0.1 %) added. The gradient used was: 0–10 minutes, 10% acetonitrile; 10–30 minutes, 10% acetonitrile to 100% acetonitrile; 30–35 minutes, 100% acetonitrile; the column was then restored to its initial state (10% acetonitrile) for 15 minutes before reuse. Absorbance was monitored at a wavelength of 324 nm. Fractions containing venemycin were identified by ESI‐MS and combined. The combined fractions were evaporated under reduced pressure, freeze‐dried, reconstituted into [D_6_]DMSO (180 μL) and transferred to a 3 mm NMR tube. ^1^H, HSQC and HMBC experiments were carried out with a Bruker Avance II 400 MHz NMR spectrometer.

**Assay of antibacterial activity**: Colonies of *M. luteus* ATCC4698 and *E. coli* ATCC25922 freshly grown on lysogeny broth (LB) agar plates were used separately to inoculate LB broth (10 mL) followed by overnight cultivation at 30 °C. Overnight culture (0.2 mL) was used to inoculate LB (10 mL) and cultivation at 30 °C was continued until an OD _600_ of 0.6–0.8 was reached. The culture (1 mL) was added to LB‐agar medium (50 mL) at about 45 °C, and 25 mL were poured into 10×10 cm plates. Culture supernatant (100 μL) was added to wells made in the solidified agar, and plates were incubated at 30 °C for 24–48 h, after which they were monitored for the appearance of inhibitory halos. Kanamycin (25 μg mL^−1^) was used as positive control.

**Abbreviations**

A: AMP‐forming domain. ACP: acyl carrier protein. AT: acyl transferase. CHS: chalcone synthase. CoA: coenzyme A. KR: ketoreductase. KS: ketosynthase. LAL: large‐ATP binding transcriptional regulator of LuxR family. MFS: major facilitator superfamily. TE: thioesterase.

## Supporting information

As a service to our authors and readers, this journal provides supporting information supplied by the authors. Such materials are peer reviewed and may be re‐organized for online delivery, but are not copy‐edited or typeset. Technical support issues arising from supporting information (other than missing files) should be addressed to the authors.

Supplementary
