# Supplementary material for: Discovery of Unusual Biaryl Polyketides by Activation of a Silent Streptomyces venezuelae Biosynthetic Gene Cluster
Source: Chembiochem. 2016 Oct 13;17(22):2189–98. doi: 10.1002/cbic.201600396 (PMC5132015; doi:10.1002/cbic.201600396)
Supplement: Supplementary file 1 — Supplementary [file CBIC-17-2189-s001.pdf]

CHEMBIOCHEM

Supporting Information

Discovery of Unusual Biaryl Polyketides by Activation of a Silent *Streptomyces venezuelae* Biosynthetic Gene Cluster

Anyarat Thanapipatsiri,[a, b] Juan Pablo Gomez-Escribano,[b] Lijiang Song,[c] Maureen J. Bibb,[b] Mahmoud Al-Bassam,[b, e] Govind Chandra,[b] Arinthip Thamchaipenet,*[a, d] Gregory L. Challis,*[c] and Mervyn J. Bibb*[b]

cbic_201600396_sm_miscellaneous_information.pdf

Supporting Information

RNA preparation from *S. coelicolor* for RT-PCR

S. coelicolor strains were grown in liquid TSB medium for 24-48 hours and the mycelium fragmented using glass homogenisers. Mycelial biomass was estimated by optical density (OD) at 600 nm and cultures were inoculated to a starting OD_{600} of 0.1. 2 ml of these cultures were harvested at 48 hours and stored using RNAprotect solution (Qiagen) according to the manufacturer's instructions. Mycelial pellets were resuspended in 200 µl of 10 mM Tris-HCl (pH 8), 1mM EDTA and 15 mM lysozyme. The re-suspended mycelium (250 µl) was incubated at room temperature for 60 minutes and added to 700 µl of precooled RNA lysis buffer containing guanidine thiocyanate (RLT buffer) supplemented with 10 µl/ml β-mercaptoethanol (Qiagen). The mixture was sonicated on ice for 3 cycles of 5 seconds on, 5 seconds off. The sonicator probe (amplitude 5 microns) was washed with 70% ethanol before and after sonication. The resulting lysate was subjected to two 700 µl phenol chloroform-isoamyl-alcohol (pH 8) extractions, applied to an RNeasy mini column (Qiagen) and RNA purified according to the manufacturer's instructions with an on-column DNaseI digestion. Approximately 10 µg of purified RNA was treated with RNase-free DNaseI (Promega) and purified using a RNeasy mini kit (Qiagen). RNA concentration and quality was assessed using a Nanodrop machine (Thermo Scientific) and gel electrophoresis. The absence of genomic DNA contamination in RNA samples was confirmed by attempting PCR using *Taq* polymerase (Qiagen) and the primers SCOHrdBF and SCOHrdBR, with 150-300 ng of template RNA. *S. coelicolor* M145 genomic DNA was used as a positive control with the PCR conditions described below.

RT-PCR

RNA (1µg) was converted to cDNA using a Maxima First Strand cDNA Synthesis Kit (Thermo Scientific) according to the manufacturer's instructions. The resulting cDNA was amplified using the primers listed in Table S1 in 25 µl with 2 µl cDNA template, dNTPs (2.5 mM each), 10 pmol of each primer, Q-solution*, 10x buffer* and Taq DNA polymerase*. Asterisks (*) indicate the material provided by Qiagen. PCR cycles used: denaturation at 94 °C for 1 minute, 30 cycles with denaturation at 94 °C for 30 seconds, annealing at 60 °C or 67 °C for 30 seconds, extension at 72 °C for 30 seconds, and a final elongation step at 72 °C for 10 minutes. PCR products were confirmed by analysing 10 µl of the PCR on a 2% agarose gel. *S. coelicolor hrdB* was amplified from genomic DNA as a positive control.

Figure S1. Analysis of the culture supernatant of *S. venezuelae* SV13 (Δ*bldM*) grown for four days in MYM-TAP medium. Left: Mass spectrum of venemycin (top) compared with the expected spectrum for the predicted molecular formula (bottom). Right: Ultraviolet-visible spectrum of the venemycin-containing peak. Bottom: Structure of venemycin.

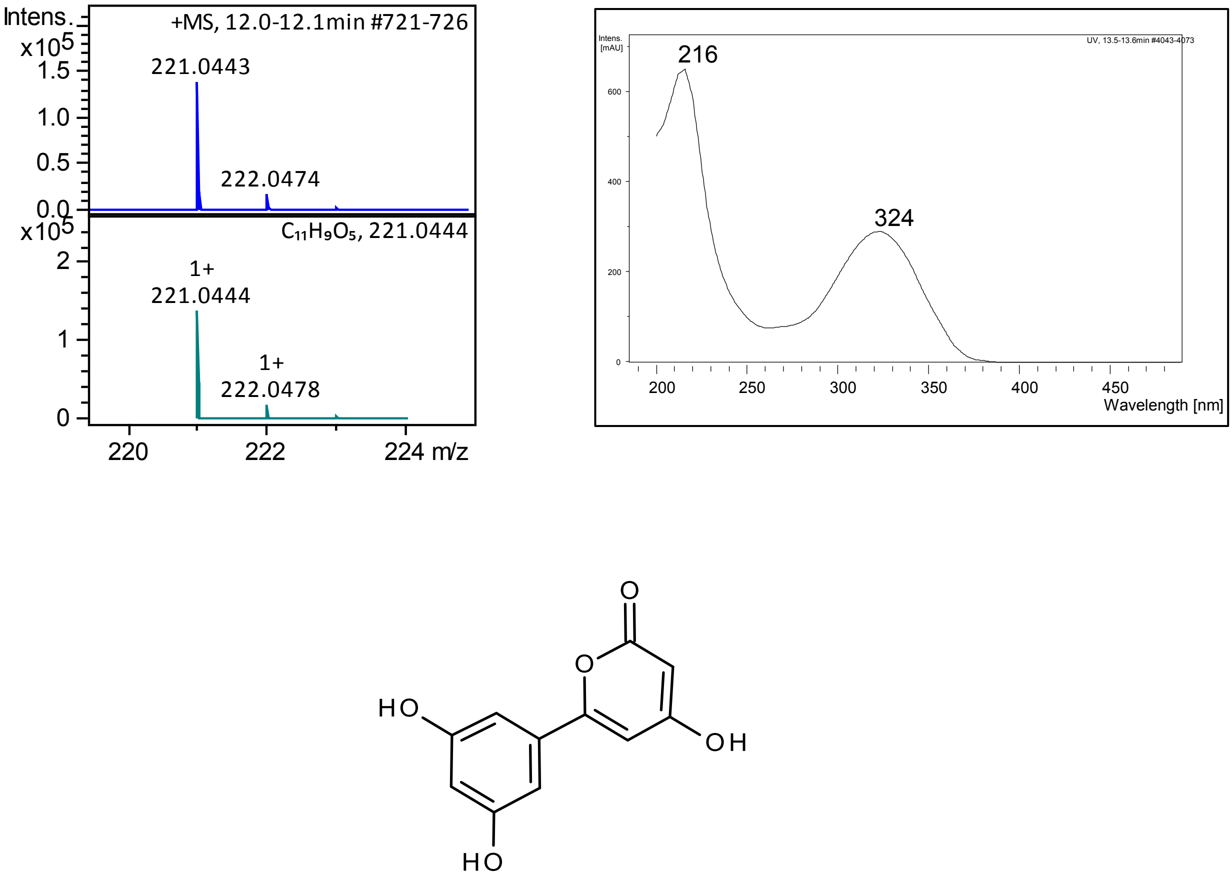

Figure S2. Top left: Extracted ion chromatogram for the expected molecular ion of singly-chlorinated venemycin showing its presence in the culture supernatant of *S. venezuelae* SV13 (Δ*bldM*) (bottom panel) but absence in that of the parental strain (top panel). The cultures were grown for four days in MYM-TAP medium. Top right: Mass spectrum of singly-chlorinated venemycin (top panel) compared with the expected spectrum for the predicted molecular formula (bottom panel). Bottom: Possible structures of singly-chlorinated venemycin derivatives.

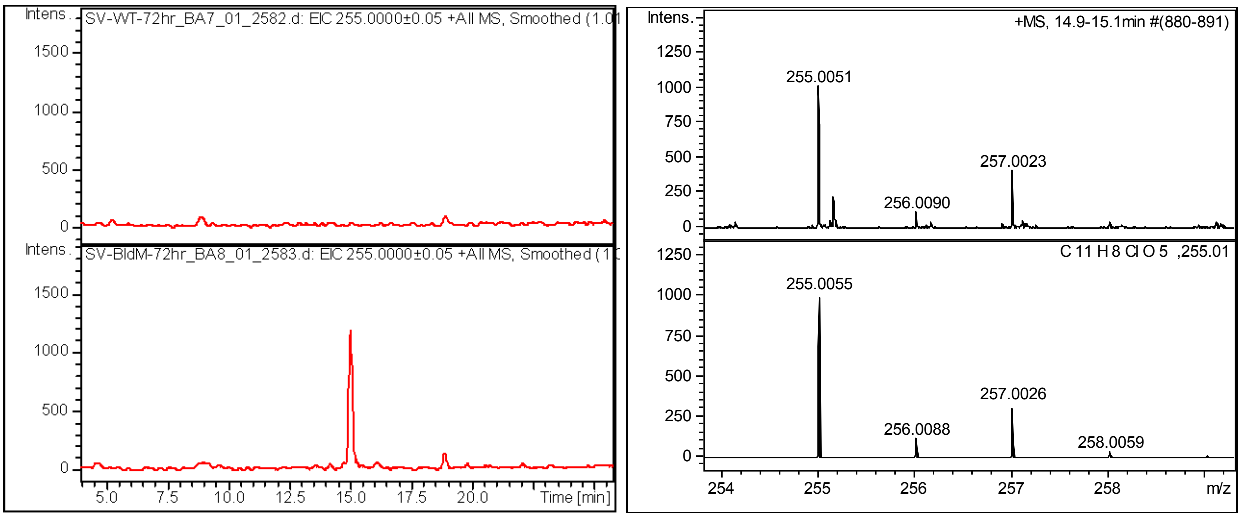

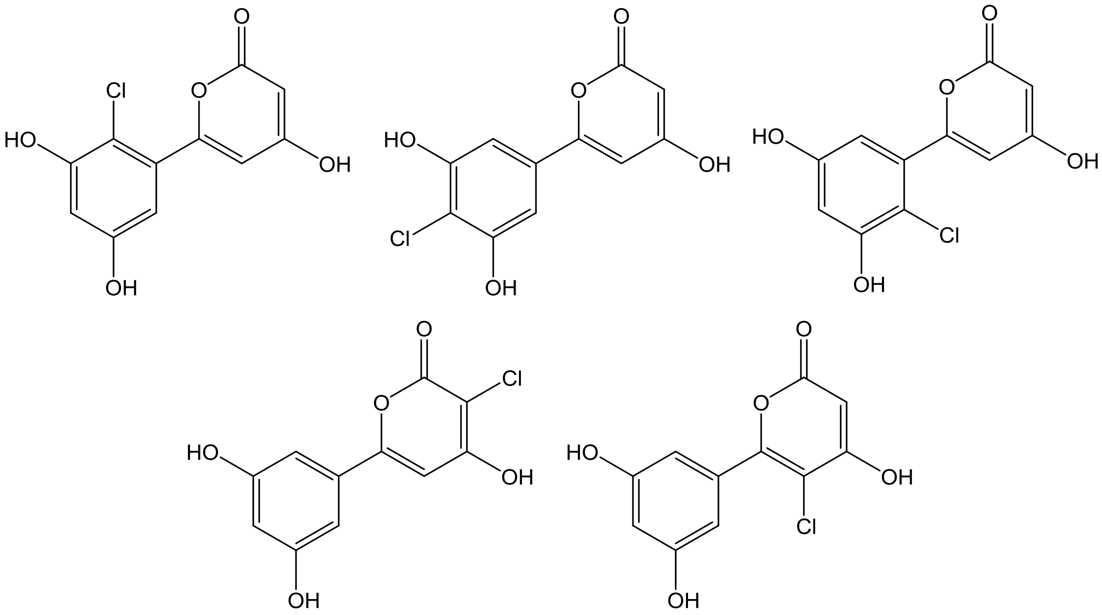

Chemical Formula: $C_{11}H_7ClO_5$
Exact Mass: 253.9982

Figure S3. ^{1}H-NMR spectrum of venemycin and co-eluted 3, 5-dihydroxybenzoic acid with an expanded version of the spectrum between 5.3 and 6.9 ppm shown below.

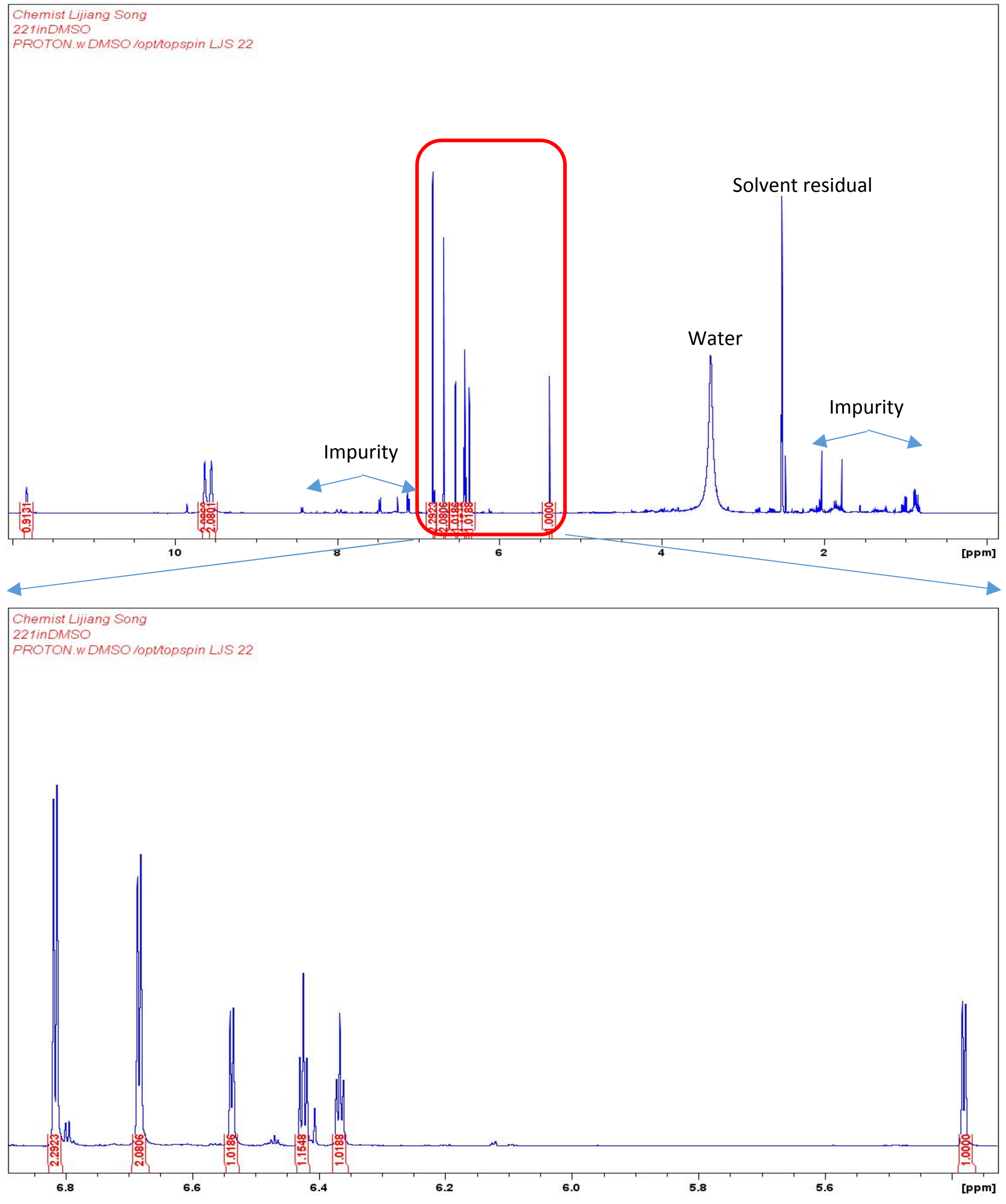

Figure S4. COSY NMR spectrum of venemycin and co-eluted 3, 5-dihydroxybenzoic acid with an expanded version of the spectrum between 5.3 and 6.9 ppm shown below.

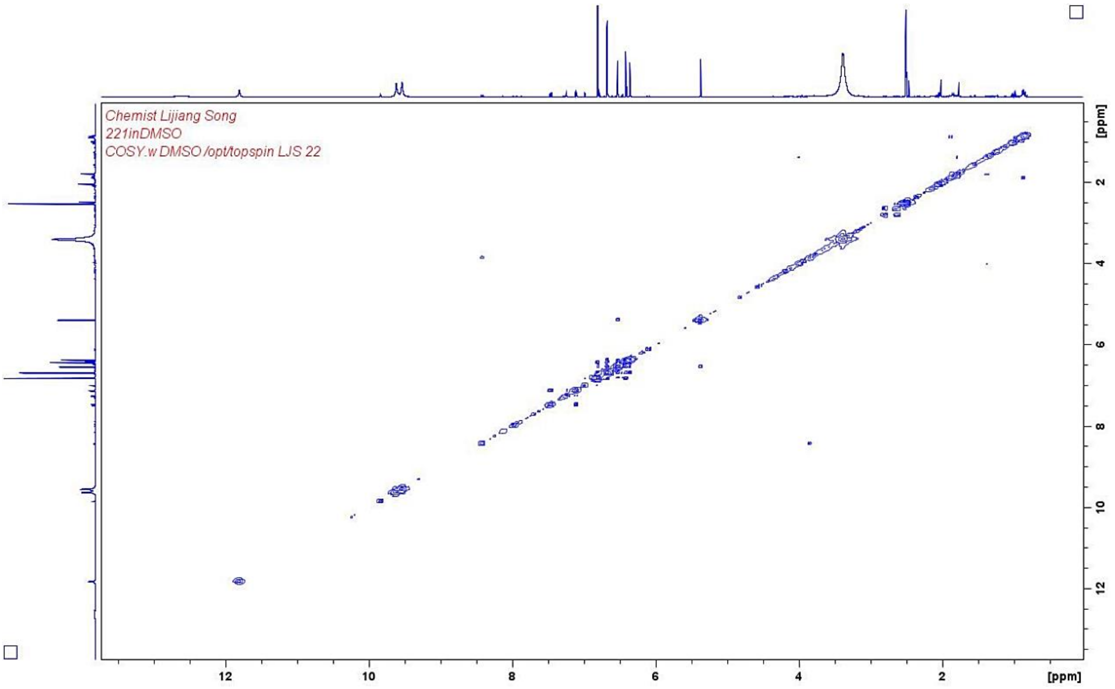

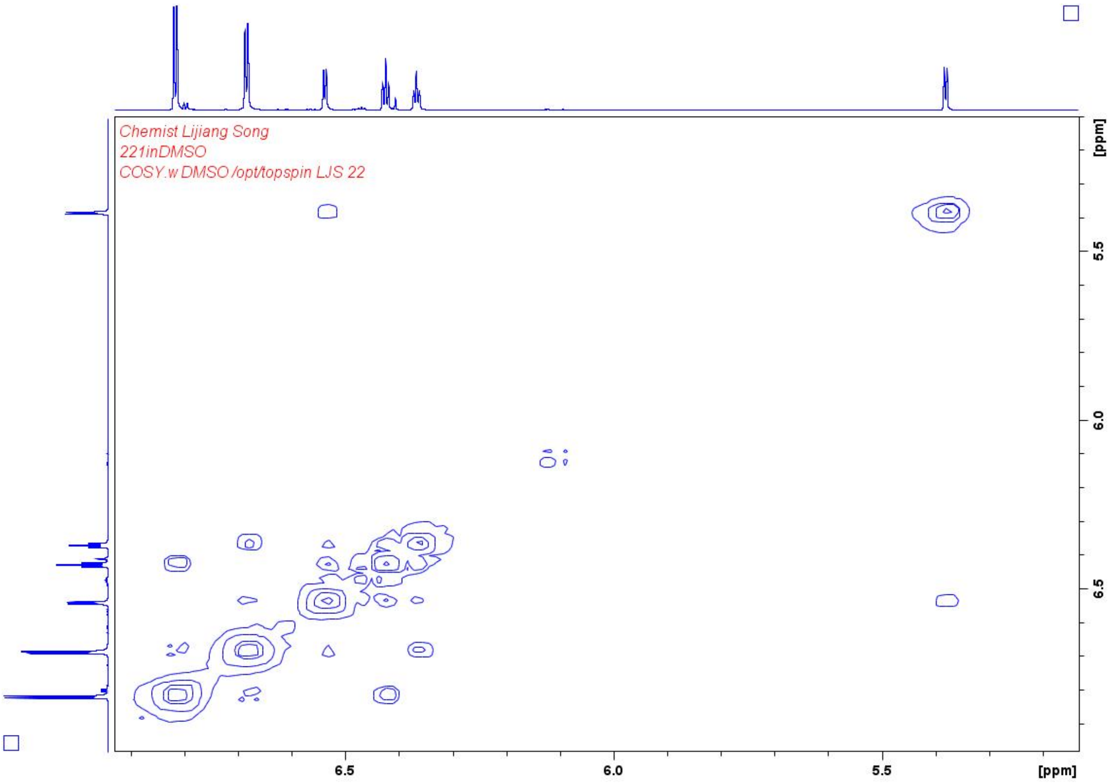

Figure S5. HSQC NMR spectrum of venemycin and co-eluted 3, 5-dihydroxybenzoic acid.

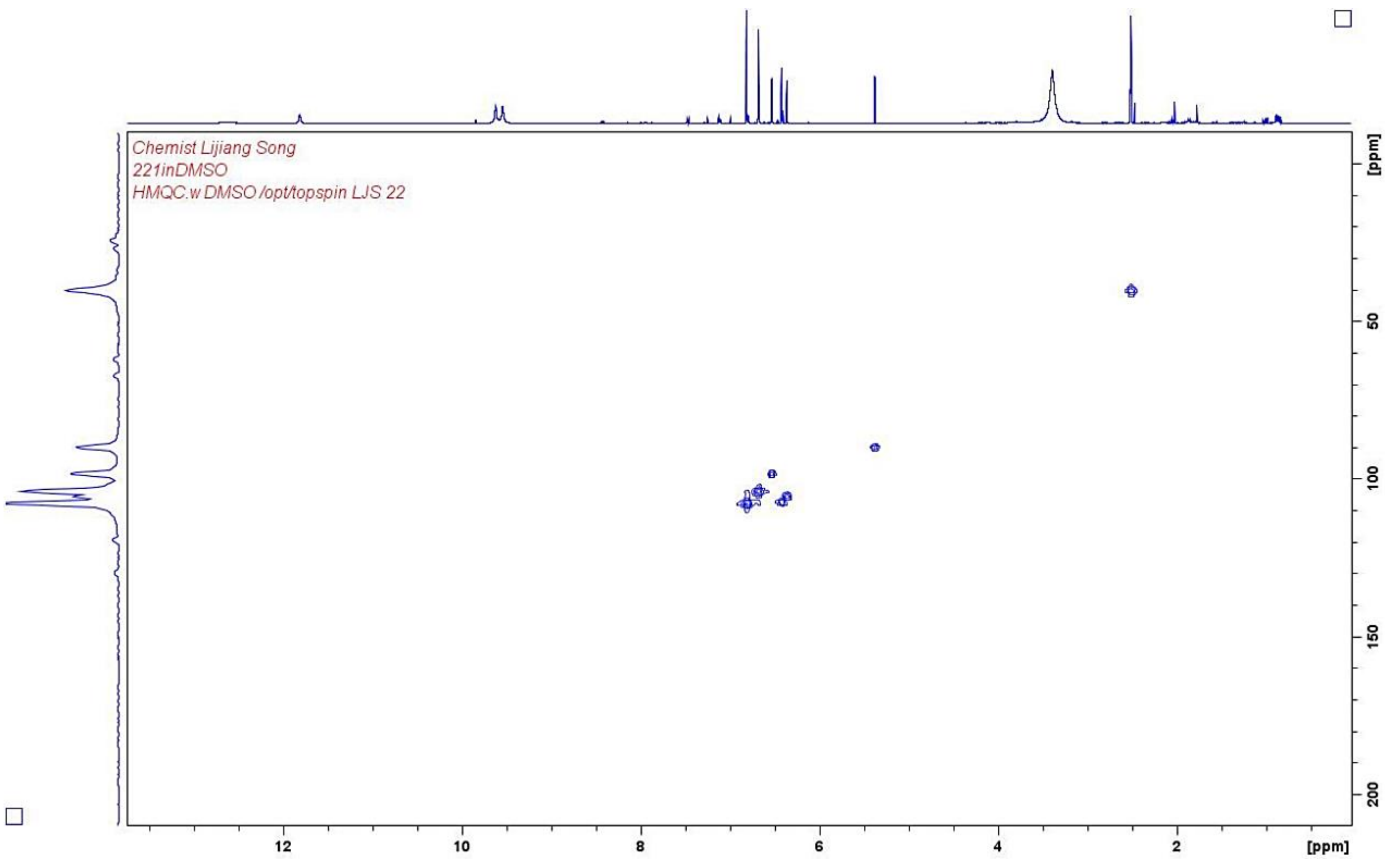

Figure S6. HMBC NMR spectrum of venemycin and co-eluted 3, 5-dihydroxybenzoic acid.

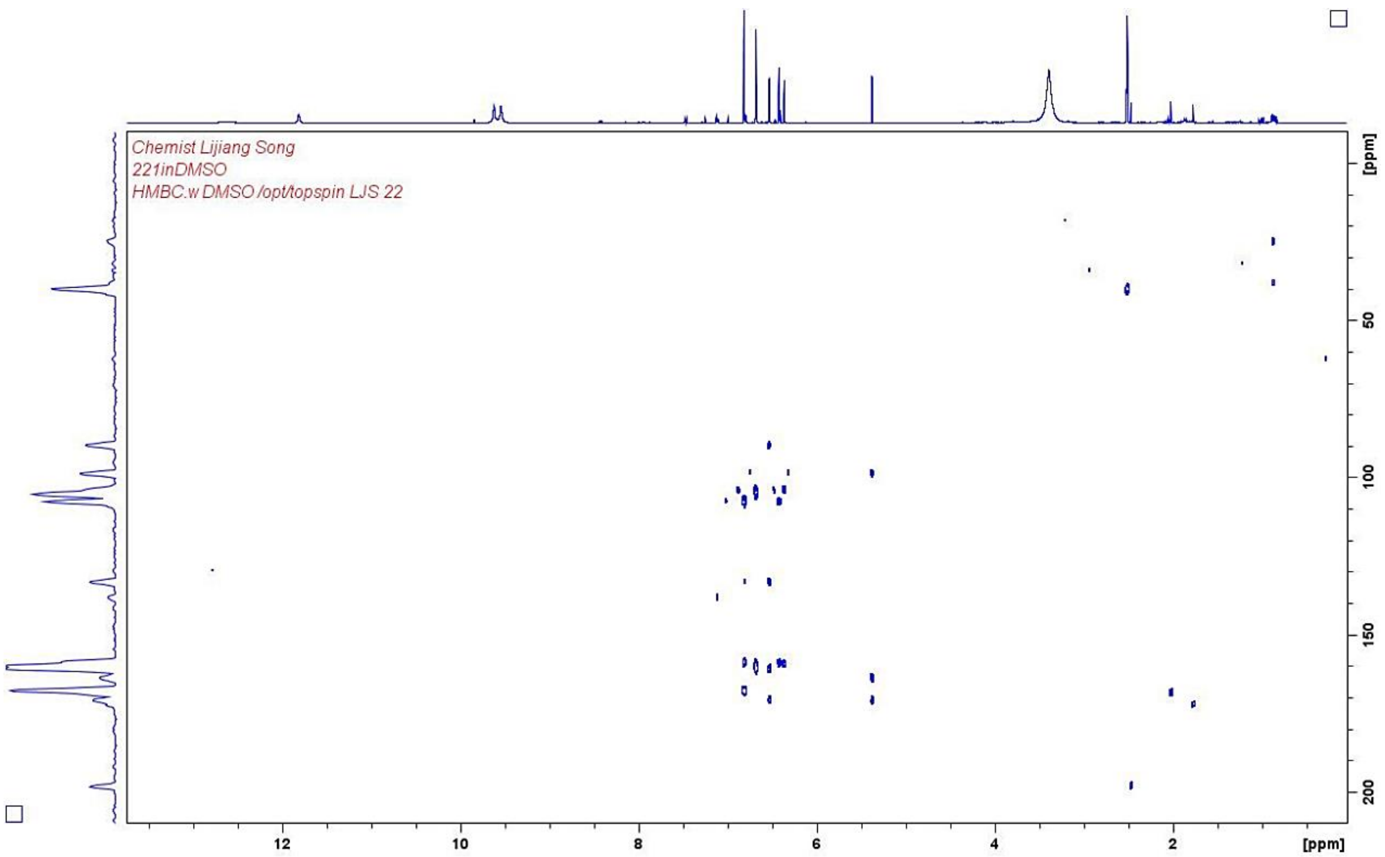

Figure S7. Structure and NMR assignments for co-eluted minor component 3, 5-dihydroxybenzoic acid (DMSO-d6, 400MHz).

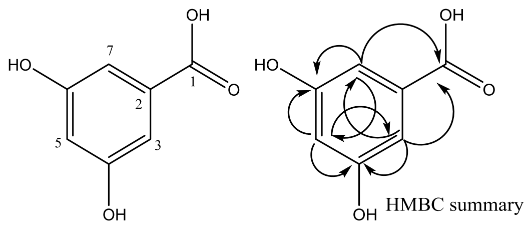

Carbon No	^{1}H (ppm, *J* Hz)	^{13}C (ppm)*	HMBC
1	11.81 (b)	167.8	
2		133.3	
3	6.82 (d, 2.3)	107.7	C1, C2, C4, C5, C6
4		159.2	
4-OH	9.56 (b)		
5	6.43(t, 2.3)	107.2	C2, C3, C6, C7
6		159.3	
6-OH	9.61 (b)		
7	6.82 (d, 2.3)	107.8	C1, C3, C5, C6

*From HSQC and HMBC

Figure S8. Incorporation of chlorine or bromine into venemycin in cultures grown for 10 days in minimal medium (MM) supplemented with the corresponding halide. Panels A to D: Culture supernatants from *S. venezuelae* ∆*bldM* derivatives; Panels E to H: Culture supernatants from *S. coelicolor* M1152 derivatives; the specific strains and halide used are given in the table below. Although MM does not support good growth of *S. venezuelae* (hence the relatively low levels of production, left panels), singly-brominated venemycin was clearly detected (panel D). In *S. coelicolor* (right panels), the incorporation of bromine was more efficient and could be detected even in the absence of the halogenase expression plasmid (panel G); in contrast, no chlorinated venemycin was observed in the absence of the expression construct (panel E). Venemycin $[M-H]^-$ = 219.0299 *m/z*), chloro-venemycin $[M-H]^-$ = 252.9909, and bromo-venemycin $[M-H]^-$ = 296.9404.

Sample	MM + 0.5 g/l of	Strain
**A**	NaCl	*S. venezuelae* M1817 (Δ*bldM* + *ermE**p::*vemR*)
**B**	NaCl	*S. venezuelae* M1831 (Δ*bldM* + *ermE**p::*vemR* + *ermE**p::*vemJKL*)
**C**	NaBr	*S. venezuelae* M1817 (Δ*bldM* + *ermE**p::*vemR*)
**D**	NaBr	*S. venezuelae* M1831 (Δ*bldM* + *ermE**p::*vemR* + *ermE**p::*vemJKL*)
**E**	NaCl	*S. coelicolor* M1822 (M1152 + *vem* cluster + *ermE**p::*vemR*)
**F**	NaCl	*S. coelicolor* M1835 (M1152 + *vem*-cluster + *ermE**p::*vemR* + *ermE**p::*vemJKL*)
**G**	NaBr	*S. coelicolor* M1822 (M1152 + *vem* cluster + *ermE**p::*vemR*)
**H**	NaBr	*S. coelicolor* M1835 (M1152 + *vem*-cluster + *ermE**p::*vemR* + *ermE**p::*vemJKL*)

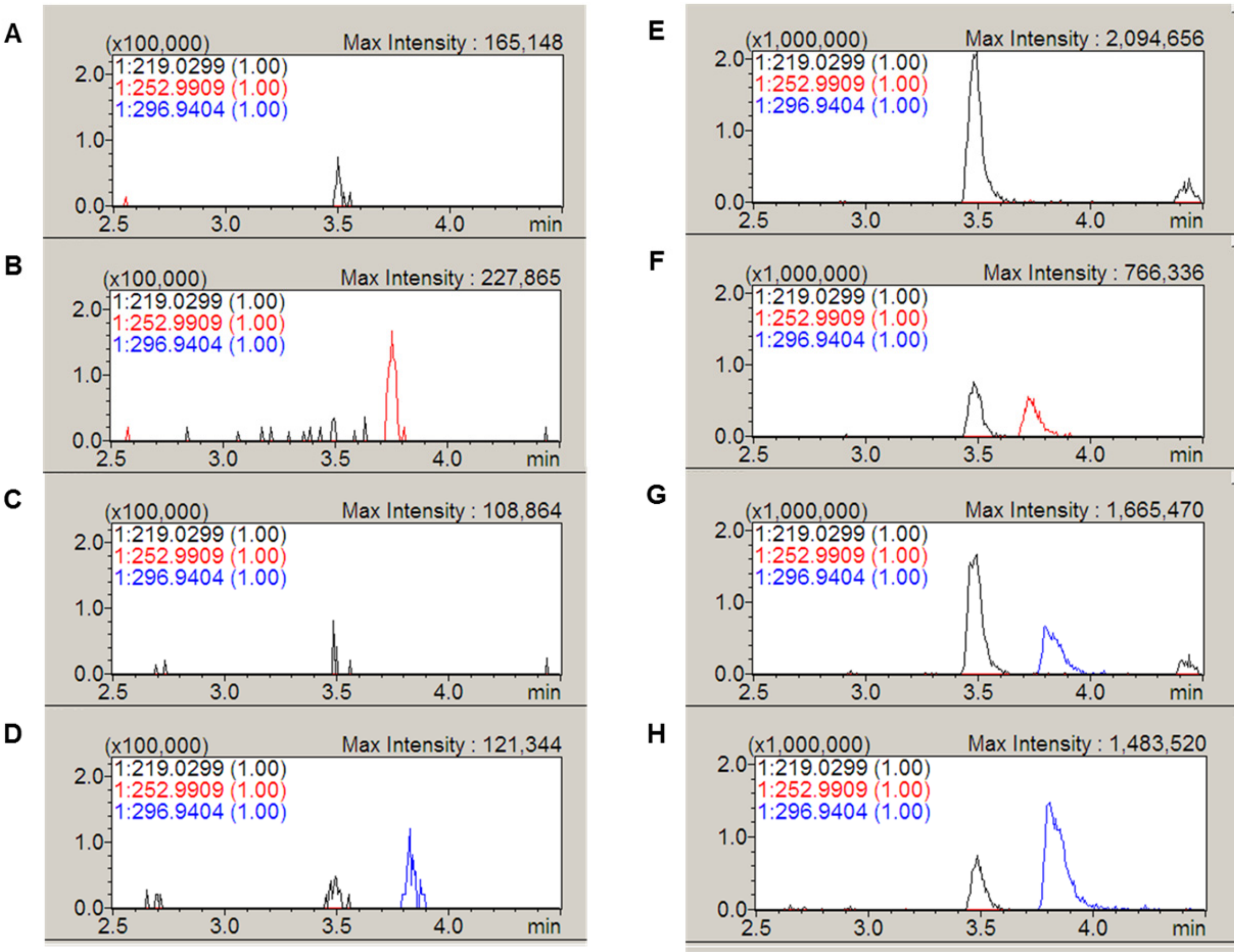
A
(x100,000)
Max Intensity : 165,148
1:219.0299 (1.00)
1:252.9909 (1.00)
1:296.9404 (1.00)
2.0
1.0
0.0
2.5
3.0
3.5
4.0
min
B
(x100,000)
Max Intensity : 227,865
1:219.0299 (1.00)
1:252.9909 (1.00)
1:296.9404 (1.00)
C
(x100,000)
Max Intensity : 108,864
1:219.0299 (1.00)
1:252.9909 (1.00)
1:296.9404 (1.00)
D
(x100,000)
Max Intensity : 121,344
1:219.0299 (1.00)
1:252.9909 (1.00)
1:296.9404 (1.00)
E
(x1,000,000)
Max Intensity : 2,094,656
1:219.0299 (1.00)
1:252.9909 (1.00)
1:296.9404 (1.00)
F
(x1,000,000)
Max Intensity : 766,336
1:219.0299 (1.00)
1:252.9909 (1.00)
1:296.9404 (1.00)
G
(x1,000,000)
Max Intensity : 1,665,470
1:219.0299 (1.00)
1:252.9909 (1.00)
1:296.9404 (1.00)
H
(x1,000,000)
Max Intensity : 1,483,520
1:219.0299 (1.00)
1:252.9909 (1.00)
1:296.9404 (1.00)

Figure S9. A) The arrow indicates the replacement of the SspI fragment containing *aph* in the SuperCos1 backbone of cosmid Sv3E02 with a 5,247 bp SspI *oriT-attP-int-aac(3)IV* fragment from pMJCOS1 (now known as pIJ10702) by PCR targeting.[1] B) The resulting cosmid pIJ13035 containing the cryptic PKS gene cluster.

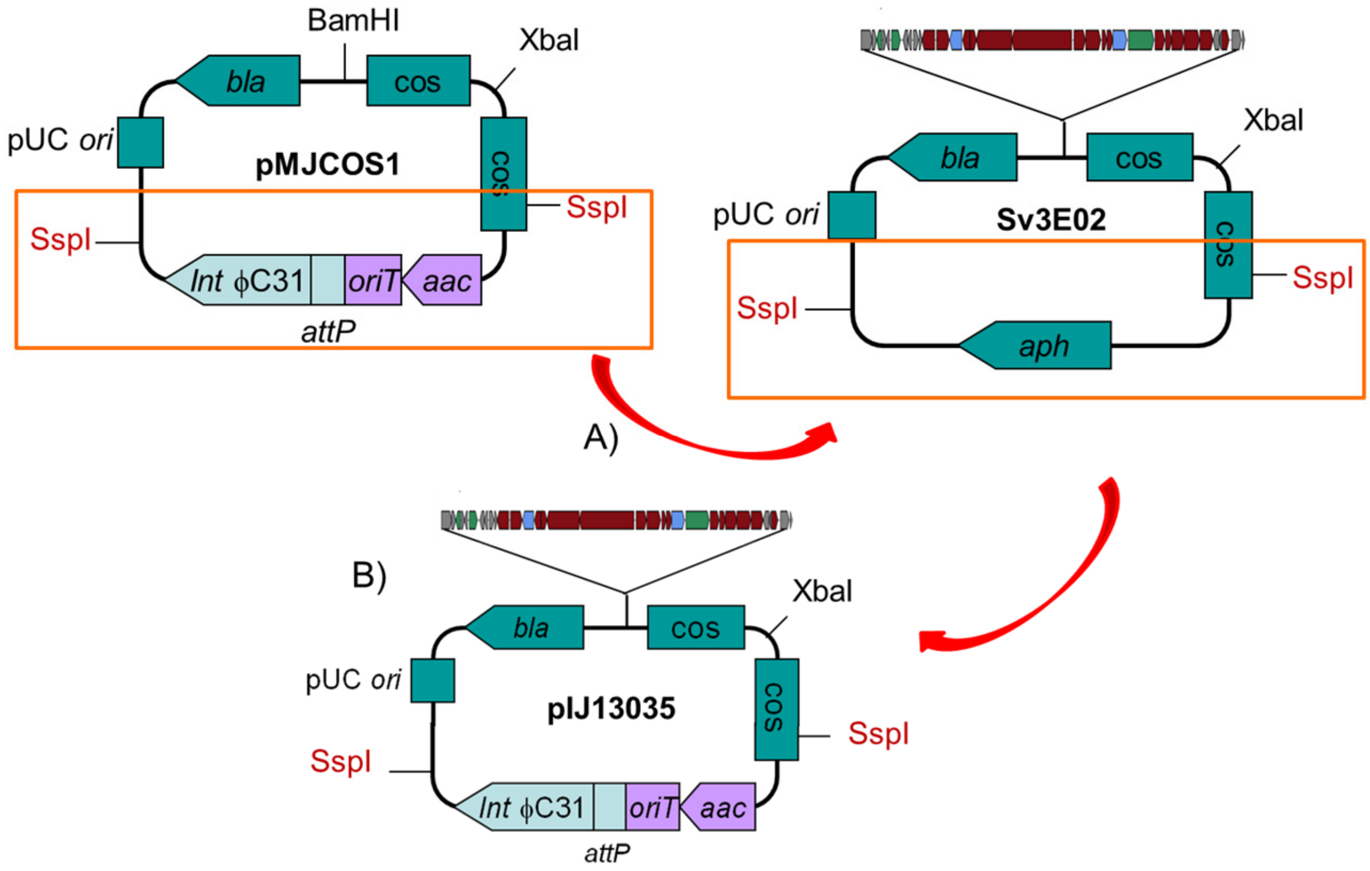

Figure S10. Schematic representation of the DNA fragments from *S. venezuelae* cosmid Sv3E02 that were cloned into pIJ10257 or pIJ12477 to create the *vemR* expression plasmid pIJ13028 and the halogenase (*vemJKL*) expression plasmid pIJ13029, respectively. *vemR* was inserted between the NdeI and HindIII sites of pIJ10257 and the halogenase cassette was inserted between the BamHI and HindIII sites of pIJ12477; in both cases, the inserted genes were placed under the control of the constitutive *ermE** promoter.

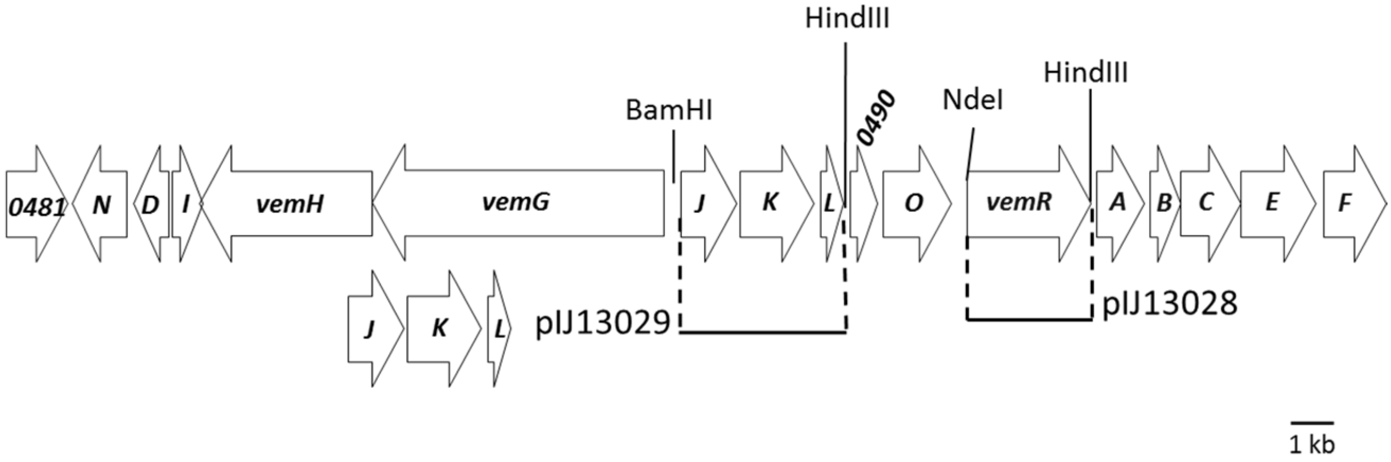

Table S1. Bacterial strains, plasmids and cosmids used and generated in this study.

Strain/Plasmid/Cosmid	Description	Source or reference
**Bacterial strains**		
Escherichia coli ATCC 25922	Bioassay indicator organism	ATCC
E. coli DH5α	*deoR recA1 endA1 hsdR17*(*rk*$^-$, *mk*$^+$) *phoA supE44 thi-1 gyrA96 relA1 λ*$^-$	Invitrogen, USA
E. coli BW25113/pIJ790	(Δ(*araD-araB*)*567*, Δ*lacZ4787* (::*rrnB-4*), *lacIp-4000*(*lacI*^{Q}), λ$^-$, *rpoS369*(Am), *rph-1*, Δ(*rhaD-rhaB*)568, *hsdR514*/ λ-Red plasmid, [*oriR101*], [*repA101*(ts)], *araBp-gam-bet-exo*	[2]
E. coli BT340	DH5α/pCP20	[3]
E. coli ET12567/pUZ8002	*dam-13::Tn9 dcm-6 hsd*M *hsd*S Km^r/ *tra, neo*, RP4	[4]
Micrococcus luteus ATCC 4698	Bioassay indicator organism	ATCC
S. coelicolor M1152	Derivative of *S. coelicolor* M145, Δ*act* Δ*red* Δ*cpk* Δ*cda rpoB* (C1298T)	[5]
S. coelicolor M1316	M1152 Δ*SCO7221* Δ*SCO7669-7670-7671*	[6]
S. coelicolor M1818	*S. coelicolor* M1152:: pIJ13035	This work
S. coelicolor M1819	*S. coelicolor* M1316:: pIJ13035	This work
S. coelicolor M1822	*S. coelicolor* M1152:: pIJ13035:: pIJ13028	This work
S. coelicolor M1825	*S. coelicolor* M1316:: pIJ13035:: pIJ13028	This work

S. coelicolor M1835	*S. coelicolor* M1152:: pIJ13035:: pIJ13028+pIJ13029	This work
S. coelicolor M1839	*S. coelicolor* M1316:: pIJ13035:: pIJ13028+pIJ13029	This work
S. venezuelae ATCC10712	Wild type strain	[7]
S. venezuelae M1815	*S. venezuelae* wild type:: pIJ13028	This work
S. venezuelae M1817	*S. venezuelae* SV13:: pIJ13028	This work
S. venezuelae M1827	*S. venezuelae* wild type:: pIJ13028+pIJ13029	This work
S. venezuelae M1831	*S. venezuelae* SV13:: pIJ13028+pIJ13029	This work
S. venezuelae SV13	Δ*bldM::oriT- aac(3)IV*	[8]
**Plasmids**		
pBlueScript II KS (+)	General cloning vector	[9]
pIJ773	pBluescript KS (+), *aac(3)IV*, *oriT* (RK2), FRT sites	[1]
pIJ790	λ-RED (*gam*, *bet*, *exo*), *cat*, *araC*, *rep101*^{ts}	[1]
pIJ10257	*oriT*, ØBT1 *int-attB*, *hyg*, *ermE*p*	[10]
pIJ12477	*ermEp**, *aac(3)IV*, *neo*, *oriT* (RK2), *ori* (pIJ101), *ori* (pUC18)	[11]
pIJ13028	pIJ10257 containing *ermE**p::*vemR*	This work
pIJ13029	pIJ12477 containing *ermE**p::*vemJKL*	This work
**Cosmids**		
pIJ13035	Sv3E02::SspI fragment (*aac(3)IV*, *oriT*, ØC31 *int-attP* from pMJCOS1)	This work

pMJCOS1	SuperCOS1, *bla*, *aac(3)IV*,	[12]
SuperCos1	*neo*, *bla*	Agilent technologies, USA
SV-3-E02	SuperCos1 containing *vem* gene cluster cluster (from nucleotide 511229 to 554231 of GenBank accession number FR845719).	John Innes Centre, UK

Table S2. Primers used in this study.

Oligomer	Sequence 5'-3'	Description/use
OSVEN04812F	ACCACGTCGTCGAGGTCAG	Intergenic region between *sven 0481* and
OSVEN04812R	TCTTCCTGCTGCTGTGCGTC	*vemN* (*sven0482*)
OSVEN04832F	CCACCCGGTACGTCTGGG	Intergenic region between *vemN* (*sven 0482*)
OSVEN04832R	GGAACTCCTGGACGCTCAGC	and *vemD* (*sven0483*)
OSVEN04854FN	CGAGCAGTGGTCACGGTCAC	Intergenic region between *vemI* (*sven 0484*)
OSVEN04854RN	GCCGACACCCTGCATCCG	and *vemH* (*sven0485*)
OSVEN04865F	CGCACGGCTCACCGAAG	Intergenic region between *vemH* (*sven 0485*)
OSVEN04865R	GCAACTGCCTGCGGGTC	and *vemG* (*sven0486*)
OSVEN04878F	ACCACAAGCTGGCGCAGTC	Intergenic region between *vemJ* (*sven 0487*)
OSVEN04878R	TGCCGAGGATCGCCACG	and *vemK* (*sven0488*)
OSVEN04889F	CGCCAAGGAGGCCGAGTC	Intergenic region between *vemK* (*sven 0488*)
OSVEN04889R	CAGATCGGCTTCGTACCGCTG	and *vemL* (*sven0489*)
OSVEN048990F	CCTGGTCACCGCGCTGAG	Intergenic region between *vemL* (*sven 0489*)
OSVEN048990R	ATGTTCAGCACCAGCAGGGC	and *sven0490*
OSVEN04901F	CGTTCAGCCACAACCTGGAG	Intergenic region between *vemM* (*sven 0490*)
OSVEN04901R	GCGGAATGGGCTTGTCG	and *vemO* (*sven0491*)
OSVEN04912F	TGGTGTTCGCCGCCAAC	Intergenic region between *vemO* (*sven 0491*)
OSVEN04912RN	TCCGCGAAGGTGTGCAGC	and *vemR* (*sven0492*)
OSVEN04923F	AGCAGCATCTGACGCAGGTC	Intergenic region between *vemR* (*sven 0492*)
OSVEN04923R	AAGGGGTCGTGCGTTCACTG	and *vemA* (*sven0493*)
OSVEN04934F	GCGACTACGGCAACCTCTCC	Intergenic region between *vemA* (*sven 0493*)
OSVEN04934R	GATGTCGAGGACGGTGTCGG	and *vemB* (*sven0494*)
OSVEN04945F	CGCTGGGCAAGCATCTCG	Intergenic region between *vemB* (*sven 0494*)
OSVEN04945R	TCCGCCGTGTACGCCATCAG	and *vemC* (*sven0495*)
OSVEN04956FN	CGACGTGATCGACAAGGTGGAC	Intergenic region between *vemC* (*sven 0495*)
OSVEN04956R	ACCATGCCGTTGGCCAGAC	and *vem* (*sven0496*)
OSVEN04967F	AGCTCGACTTCACACGGCTC	Intergenic region between *vemE* (*sven 0496*)

OSVEN04967R	CGCCATCCGTCGCTGAACAG	and *vemF* (*sven0497*)
SCOhrdBF	CGAGTCCGTCTCTGTCATGG	*hrdB* for control for gDNA contamination
SCOhrdBR	CATCAGCGTCACACCCTCTT	
pIJ86F1	ACGCCTGGTCGATGTCGGAC	Sequencing primers for pIJ12477 derivatives
pIJ86R2	TGCGGTCAGTGCGTGTGTCG	
SVEN0487BamHIF	AAAAAA**GGATCC**TCTCGGCACGTTTCGGATCG	PCR of *vemJKL* for cloning into pIJ12477
SVEN0489HindIIR	AAAAAA**AAGCTT**CCGCCGTCAGGAGATGAAGTC	
SVEN0487TF	AGGCGTTCAGCCCGTCTCGTCGAACC	Primers for verifying integration of *vem* gene cluster in *S. coelicolor* hosts
SVEN0487TR	GACAGCGCCTTCAGCTCGGGCAC	
SVEN0493TF	GGGTCTCCAGTGTTCCGCATTCCGTG	
SVEN0493TR	CACAGCGGACGGGTGCTGTCGAT	
SVEN0487W1	ACAAGGGCACGGTCAGCG	Sequencing primers for confirmation of *vemJKL* expression constructs
SVEN0488W1	ACCAGCCGAGTCCGTGG	
SVEN0488W2	TCGGCTTCGGGCACTACG	
SVEN0489W1	AGTGAGGACCCATGTCGCAC	
SVEN0492NdeIF1	AAAAAA**CATATG**GTGCTGGTCGAGCGGGAGCGTC	PCR of *vemR* (*sven0492*) for cloning into pIJ10257
SVEN0492HindIIIR	AAAAAA**AAGCTT**CCTCCTTCTGTGAAGGTCGTCACCGG	
SVEN0492NW1	TCAGCGACCAGGACGGC	Sequencing primers for cloned *vemR* (*sven0492*)
SVEN0492NW2	CGAACACCTGCTGTGGCAC	
SVEN0492W3	GGCTCGCCAGGACGGAATCC	
SVEN0492W4	TGGAGACGCCGTTCGGGC	
SVEN0492NW5	TGGAGACGCCGTTCGGG	

Note: Bold type indicates restriction sites. The underline indicates the presence of the *vemR* ATG start codon in the enzyme restriction site used for cloning.

Table S3 NMR assignments (DMSO-d6, 400MHz) for Figure 4.

Carbon No	^{1}H (ppm, *J* Hz)	^{13}C (ppm)*	HMBC
1		170.9	
2	5.37 (d, 2.1)	89.7	C1, C3, C4
3		163.6	
3-OH	11.81 (b)		
4	6.54 (d, 2.1)	98.4	C2, C3, C5, C6
5		160.7	
6		132.9	
7	6.68 (d, 2.2)	104.1	C5, C6, C8, C9, C11
8		160.3	
8-OH	9.56 (b)		
9	6.36 (t, 2.2)	105.4	C7, C8, C10, C11
10		160.4	
10-OH	9.61 (b)		
11	6.68 (d, 2.2)	104.1	C5, C6, C7, C9, C10

*From HSQC and HMBC

References

[1] B. Gust, G. Chandra, D. Jakimowicz, T. Yuqing, C. J. Bruton, K. F. Chater, *Adv. Appl. Microbiol.* **2004**, *54*, 107-128.

[2] K. A. Datsenko, B. L. Wanner, *Proc. Natl. Acad. Sci. USA* **2000**, *97*, 6640-6645; bB. Gust, G. L. Challis, K. Fowler, T. Kieser, K. F. Chater, *Proc. Natl. Acad. Sci. USA* **2003**, *100*, 1541-1546.

[3] P. P. Cherepanov, W. Wackernagel, *Gene* **1995**, *158*, 9-14.

[4] D. J. MacNeil, K. M. Gewain, C. L. Ruby, G. Dezeny, P. H. Gibbons, T. MacNeil, *Gene* **1992**, *111*, 61-68; bM. S. Paget, L. Chamberlin, A. Atrih, S. J. Foster, M. J. Buttner, *J Bacteriol* **1999**, *181*, 204-211.

[5] J. P. Gomez-Escribano, M. J. Bibb, *Microb. Biotechnol.* **2011**, *4*, 207-215.

[6] A. Thanapipatsiri, J. Claesen, J. P. Gomez-Escribano, M. Bibb, A. Thamchaipenet, *Microb. Cell Fact.* **2015**, *14*, 145.

[7] J. Ehrlich, D. Gottlieb, et al., *J. Bacteriol.* **1948**, *56*, 467-477.

[8] M. M. Al-Bassam, M. J. Bibb, M. J. Bush, G. Chandra, M. J. Buttner, *PLoS Genet.* **2014**, *10*, e1004554.

[9] M. A. Alting-Mees, J. M. Short, *Nucleic Acids Res.* **1989**, *17*, 9494.

[10] H. J. Hong, M. I. Hutchings, L. M. Hill, M. J. Buttner, *J. Biol. Chem.* **2005**, *280*, 13055–13061.

[11] J. Claesen, *PhD thesis*, University of East Anglia **2010**.

[12] K. Yanai, T. Murakami, M. Bibb, *Proc. Natl. Acad. Sci. USA* **2006**, *103*, 9661-9666.
